# Quantitative softness and texture bimodal haptic sensors for robotic clinical feature identification and intelligent picking

**DOI:** 10.1126/sciadv.adp0348

**Published:** 2024-07-24

**Authors:** Ye Qiu, Fangnan Wang, Zhuang Zhang, Kuanqiang Shi, Yi Song, Jiutian Lu, Minjia Xu, Mengyuan Qian, Wenan Zhang, Jixuan Wu, Zheng Zhang, Hao Chai, Aiping Liu, Hanqing Jiang, Huaping Wu

**Affiliations:** ^1^ College of Mechanical Engineering, Key Laboratory of Special Purpose Equipment and Advanced Processing Technology, Zhejiang University of Technology, Hangzhou, Zhejiang 310023, China.; ^2^ School of Engineering, Westlake University, Hangzhou, Zhejiang 310030, China.; ^3^ College of Information Engineering, Zhejiang University of Technology, Hangzhou, Zhejiang 310023, China.; ^4^ Zhijiang College of Zhejiang University of Technology, Shaoxing, Zhejiang 312030, China.; ^5^ Center for Optoelectronics Materials and Devices, Zhejiang Sci-Tech University, Hangzhou, Zhejiang 310018, China.; ^6^ Collaborative Innovation Center of High-end Laser Manufacturing Equipment (National “2011 Plan”), Zhejiang University of Technology, Hangzhou, Zhejiang 310023, China.

## Abstract

Replicating human somatosensory networks in robots is crucial for dexterous manipulation, ensuring the appropriate grasping force for objects of varying softness and textures. Despite advances in artificial haptic sensing for object recognition, accurately quantifying haptic perceptions to discern softness and texture remains challenging. Here, we report a methodology that uses a bimodal haptic sensor to capture multidimensional static and dynamic stimuli, allowing for the simultaneous quantification of softness and texture features. This method demonstrates synergistic measurements of elastic and frictional coefficients, thereby providing a universal strategy for acquiring the adaptive gripping force necessary for scarless, antislippage interaction with delicate objects. Equipped with this sensor, a robotic manipulator identifies porcine mucosal features with 98.44% accuracy and stably grasps visually indistinguishable mature white strawberries, enabling reliable tissue palpation and intelligent picking. The design concept and comprehensive guidelines presented would provide insights into haptic sensor development, promising benefits for robotics.

## Teaser

A bimodal haptic sensor for quantitatively measuring softness and texture has been developed for robotic dexterous manipulation.

## INTRODUCTION

Somatosensory networks provide precise sensory feedback through cutaneous mechanoreceptors in which slow adaptive (SA) and fast adaptive (FA) receptors respond sensitively and selectively to static and dynamic pressure, enabling human hands to realize object recognition (e.g., softness and texture) for dexterous manipulation in grasping tasks (*1*–*5*). The understanding of human haptic perception has promoted advancements in robotics, endowing robots with sophisticated haptic sensing capabilities for environment awareness, recognition of threats, and fine motor tasks, thus benefiting applications in artificial intelligence, medical diagnostics, agriculture picking, and human-robot interaction (*6*–*12*). Inspired by the functionality of SA and FA receptors, several strategies have been implemented to integrate flexible haptic sensors, such as piezoresistive (*13*–*17*), piezoelectric (*18*–*21*), triboelectric (*22*, *23*), and capacitive (*24*–*27*) types, into robotic systems, enabling touch-based object recognition through the extraction and analysis of haptic information (e.g., pressure, vibration, and strain). However, achieving precise execution of adaptive grasping requiring high dexterity in robotic systems remains a daunting challenge due to the difficulty in determining the adaptive gripping force for objects with distinct softness and texture features.

Recent efforts behind this unmet need have led to some methods for the recognition of softness and texture separately (*28*–*32*). To estimate softness, sensory systems (*33*–*37*) such as self-locked stretchable strain sensors (*38*) and needle-based modulus probes (*39*) were developed, relying on static (force-displacement) and dynamic (amplitude, phase, and frequency) principles, respectively. Yet, static measuring techniques risk damaging fragile samples resulting from the same contact force, whereas dynamic methods encounter accuracy problems caused by the amplification of mass effects at high frequencies. Furthermore, their attempts concentrate only on feedback from normal stimuli, ignoring the interference caused by sensor tilt on the sample surface due to shear forces in practical application circumstances. To recognize surficial textures (*40*–*47*), artificial sensory systems based on an iontronic sensor (*48*) and integrated triboelectric-piezoresistive sensing modules (*40*) were created to effectively perceive static pressure and dynamic vibration signals. However, the sliding shear force and the interfacial slippage were neglected, leading to a failure to extract quantitative parameters in the recognition process (*43*, *49*). Thus far, the current methods are incapable of simultaneously quantifying softness and texture, primarily due to the difficulty of synergistically decoding the multidimensional static pressotactile and the dynamic vibrotactile encoding of features. Hence, designing haptic sensors capable of accurately measuring multidimensional static and dynamic stimuli is of considerable importance toward developing a nondestructive and precise methodology for assessing softness and texture features, greatly benefiting potential applications in robotic grasping tasks that require adaptive gripping force for scarless, antislippage interactions.

**Fig. 1.**
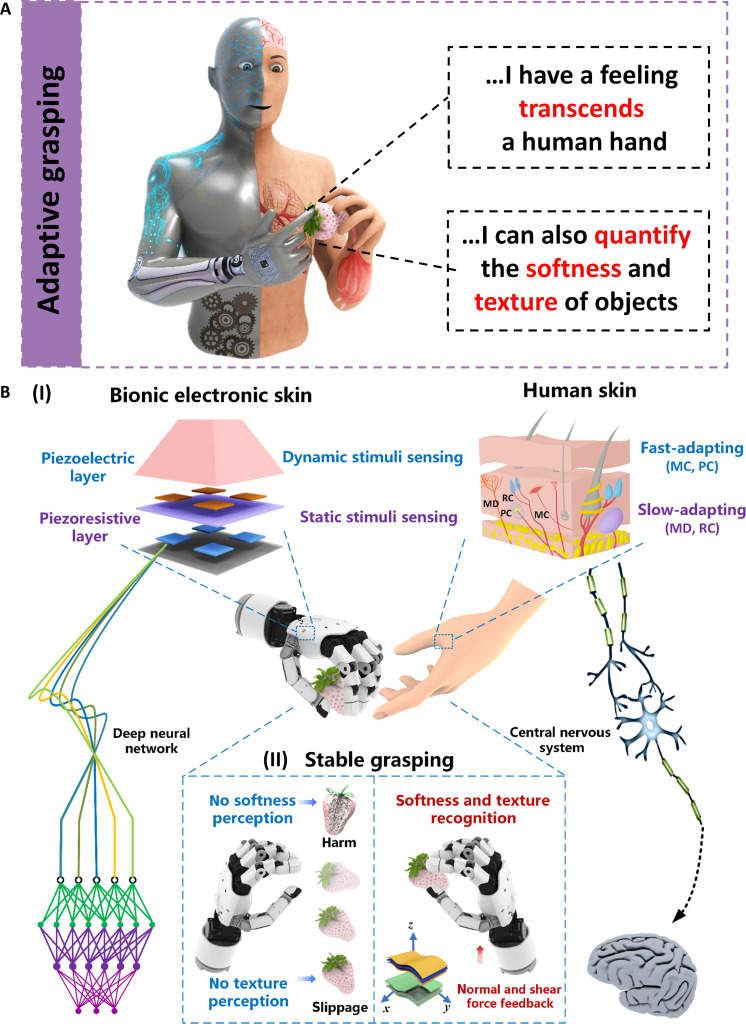
Human fingertip–inspired softness and texture bimodal haptic sensor. (**A**) Schematic illustrations of intelligent robots with anthropomorphic perception functions for quantifying the softness and texture of objects involved in the adaptive grasping process. (**B**) Schematic illustrations of the structure of human skin and the bimodal haptic sensor in an exploded view. (I) Human skin consists of slow-adapting mechanoreceptors [Merkel disc (MD) and Ruffini corpuscle (RC)] for static stimuli and fast-adapting mechanoreceptors [Meissner corpuscle (MC) and Pacinian corpuscle (PC)] for dynamic stimuli. When the fingertip touches an object, excitatory potentials are generated and transmitted along the sensory synapse to the brain, allowing the evaluation of the softness and texture characteristics of the object. The haptic sensor comprises two flexible sensing layers for piezoelectric and piezoresistive modes: The piezoelectric layer mimics the fast-adapting mechanoreceptors to sense high-frequency dynamic stimuli, while the piezoresistive layers capture static stimuli like the slow-adapting mechanoreceptors. The artificial haptic sensor is capable of simultaneously identifying softness and texture characteristics using multidimensional piezoresistive and piezoelectric feedback mechanisms and with the assistance of the depth network. (II) The bimodal sensor, designed for dynamic and static three-dimensional force perception, outperforms traditional sensors lacking softness and texture feedback, thus enabling stable, damage-free handling of delicate white strawberries without slippage.

Here, we report a quantitative softness and texture bimodal haptic sensor that transcends human haptic perception for implementation in adaptive grasping tasks of intelligent robots (Fig. 1A). The synergistic effect of piezoelectric and piezoresistive modules that mimic the functionality of FA and SA receptors within biological perception (Fig. 1B, I) allows for the detection of both static pressure and high-frequency vibrations to further identify softness and texture characteristics assisted by a deep neural network similar to the central nervous system. The multidimensional sensing capabilities enable precise elastic coefficient measurements regardless of the interaction orientation while also decoding texture-induced deformation and interfacial adhesion. The robot integrated with this bimodal haptic sensor is capable of performing challenging tasks such as identifying porcine esophageal pathological tissues and adaptively grasping white strawberries with an appropriate gripping force without damage or slippage (Fig. 1B, II). Our work presents a methodology toward quantitative, nondestructive, and precise softness and texture measurement platforms adaptable to various scenarios, which would open up many applications including clinical diagnosis, artificial intelligence, and human-machine interactions.

## RESULTS

### Sensing characterization of the bimodal multidimensional sensor

**Fig. 2.**
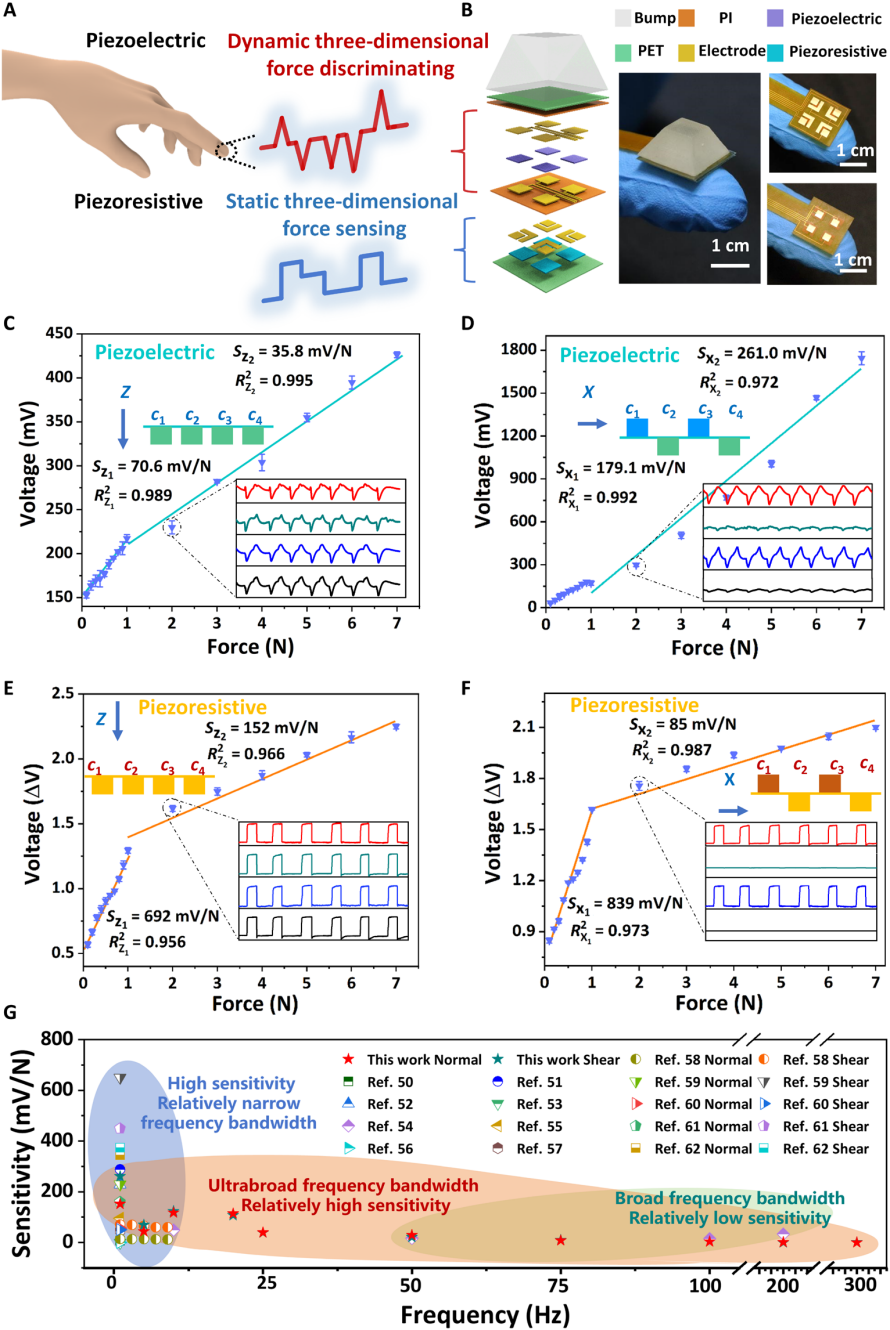
Structure, sensing principle, and multidimensional force sensing performance of the bimodal haptic sensor. (**A**) Design concept of the bimodal haptic sensor. (**B**) Detailed structure and optical images of the bimodal haptic sensor for dynamic and static multidimensional force sensing, including the top bump-shaped layer that transmits the stimuli, the sensory piezoelectric and piezoresistive layers, and electrode layers. The sensitivity of bimodal sensors based on (**C** and **D**) piezoelectric and (**E** and **F**) piezoresistive mechanisms varies when the applied normal and shear forces range from 0.1 to 7 N. Insets show the measured voltages from piezoelectric and piezoresistive modules under a loading force of 2 N. The error bars represent ±SD from the mean value (*n* = 3). (**G**) Comparison of the sensitivity of our bimodal haptic sensor with existing tactile sensors (*50*–*62*).

To enable multiple mechanical force sensing functionalities, our haptic sensor is designed with piezoelectric and piezoresistive layers (Fig. 2A), allowing for the simultaneous measurement of static and dynamic three-dimensional force information. Specifically, the piezoelectric layer [poly(vinylidene fluoride-*co*-trifluoroethylene), P(VDF-TrFE); 3 mm × 3 mm × 28 μm] presents advantages in dynamic high-frequency stimuli (≤1100 Hz) detection and ultrafast response time (fig. S1), whereas the piezoresistive layer (composite of carbon nanotubes and silicone rubber; 3 mm × 3 mm × 102 μm) is capable of detecting static pressure (fig. S2). To realize multidimensional force perception, a top bump-shaped layer made of soft polymer is further introduced into one sensory unit (Fig. 2B), efficiently transmitting external stimuli to the inner structure. The sensory layers are attached with patterned electrodes, which are used to receive the stimuli from the top force-transmission layer and convert them into electrical signals. In the presented examples here, the three-dimensional force sensing principle of the piezoelectric sensing module is further studied through finite element analysis (FEA) while applying the normal and shear forces. FEA results (fig. S3A) demonstrate that the top bump-shaped layer experiences uniform compression deformation under a uniaxial normal force along the *z* direction, resulting in four distributed piezoelectric modules (i.e., *c*_1_ to *c*_4_) with a similar piezoelectric potential of 240 mV. Considering that a shear force is applied along the *x* direction, it leads to an asymmetrical potential in the piezoelectric layer, gradually ranging from −300 to 350 mV across the arrays (fig. S3B). We then develop an analytical decoupling model (text S1) to estimate the three-dimensional force applied on the sensor through the output voltages measured on the four separated sensing units.

Responses of each sensing element in array configurations yield separate electrical outputs that allow independent characterization and decoupling of the applied force. When the haptic sensor with integrated bump (upper and bottom side lengths of 13 and 6 mm, respectively; height of 7.5 mm) is subjected to a normal force of 1 Hz, uniform compressive deformations at four distributed piezoelectric elements (i.e., *c*_1_ to *c*_4_) yield similar strain-induced changes in piezoelectric voltages (inset of Fig. 2C), consistent with FEA results. Real-time voltage output reveals that the piezoelectric module can maintain a high sensitivity of 70.6 mV N^−1^ up to 1 N (Fig. 2C). As the normal force increases from 1 to 7 N, sensitivity considerably decreases to 35.8 mV N^−1^. This decrease is due to the further separation between the positive and negative charge carrier centers, which can only orient in a single direction, leading to the upper limit in the pressure range. A uniaxial shear force of 1 Hz applied along the *x* direction induces compressive strain and a corresponding increase in piezoelectric voltage at *c*_1_ and *c*_3_ elements while having negligible effects on the strain and voltage output of the *c*_2_ and *c*_4_ elements (inset of Fig. 2D). The piezoelectric module exhibits excellent sensitivity and good linearity toward shear force ranging from 0.1 to 7 N, with respective values of 179.1 and 261 mV N^−1^ (Fig. 2D). Besides, the piezoresistive module relies on the pressure-induced conductive pathway change, resulting in a sensitivity of 692 mV N^−1^ below a normal force of 1 N, whereas sensitivity recorded at the force range of 1 to 7 N is 152 mV N^−1^ (Fig. 2E). The shear force response capability of the piezoresistive module yields a large resistance change with a high sensitivity of 839 mV N^−1^, which becomes saturated at a sensitivity of 85 mV N^−1^ (Fig. 2F).

The negligible piezoresistive sensing signals from the *c*_2_ and *c*_4_ elements are due to the specific structural design and assembly method. The piezoresistive layer was connected to the interdigital electrode without adhesive, merely forming a contact. This setup confines the force within the polyimide (PI) layer and prevents efficient force transfer to the piezoresistive layer. This observation aligns well with the FEA results (fig. S4). We measured the sample-to-sample variation in the sensor and found that various sensors exhibit excellent consistency and negligible measurement discrepancies (the error bars are presented in Fig. 2, C to F). These measuring evaluations indicate that the haptic sensor is capable of effectively perceiving three-dimensional forces.

To further evaluate the dynamic and static multidimensional force sensing capabilities, the bimodal haptic sensor, integrated within a vibration test system, was characterized using a sinusoidal force of 1 to 7 N in the 5- to 300-Hz range. The measured outputs demonstrate its favorable frequency response and efficient differentiation of three-dimensional forces over a wide range of frequencies (fig. S5). Compared to the standard force values obtained from the testing system, this sensor achieves discrimination accuracies of 92.8% for measuring normal force and 92.9% for detecting shear force (fig. S6). Together, the bimodal multidimensional sensor exhibits high sensitivity (0.52 to 261 mV N^−1^) for stimuli ranging from 1 to 7 N across a wide frequency range (0 to 300 Hz). Comparative analysis reveals that our sensor demonstrates an ultrabroad frequency bandwidth with high sensitivity (Fig. 2G and table S1), outperforming the tactile sensors reported in the literature (*50*–*62*). In addition, it provides excellent pressure resolution (0.01 N) and a broad pressure range (0.01 to 35 N; fig. S7). Repetitive compression/release tests over 1000 cycles with normal and shear forces of 1 N each were performed, and the sensor exhibits no signal drift or fluctuation during these cyclic tests (fig. S8). Furthermore, the investigation into the long-term stability of the bimodal sensor reveals that it consistently maintained a constant electrical output after 60 days, suggesting high long-term stability under pressure (fig. S9). The aforementioned merits clearly demonstrate the substantial potential of our sensors for various applications, including health monitoring, wearable sensors, and intelligent robotics.

In actual sensing applications, sensors frequently encounter the simultaneous coupling of normal and shear forces, necessitating enhanced decoupling capabilities (*63*–*67*). We evaluated the decoupling performance of the sensor under different directions of force loading (i.e., 10°, 40°, and 70°) and found that the bimodal haptic sensor exhibits a good decoupling accuracy of 90.5% (fig. S10). Meanwhile, it is essential to have the capability to detect and differentiate multidimensional forces while experiencing variable temperature conditions and mechanical deformation. The importance of this feature originates from the fact that the configuration of integrated surfaces and variations in external environmental temperatures often affect the response of sensing devices. Here, an increase in temperature leads to an enhancement in the signal output (fig. S11), which is attributed to the increased dipole moments and charge mobility. The bimodal haptic sensor would, therefore, need to be calibrated or temperature compensated to eliminate temperature effects when it is used beyond room temperature. In addition, the bimodal haptic sensor maintains a consistent signal and a good decoupling accuracy (i.e., 86.9%) when mounted on varied surfaces (figs. S12 and S13), indicating the capability to adapt to different mechanical deformations.

### Softness measurement strategy combining dynamic and static principles

**Fig. 3.**
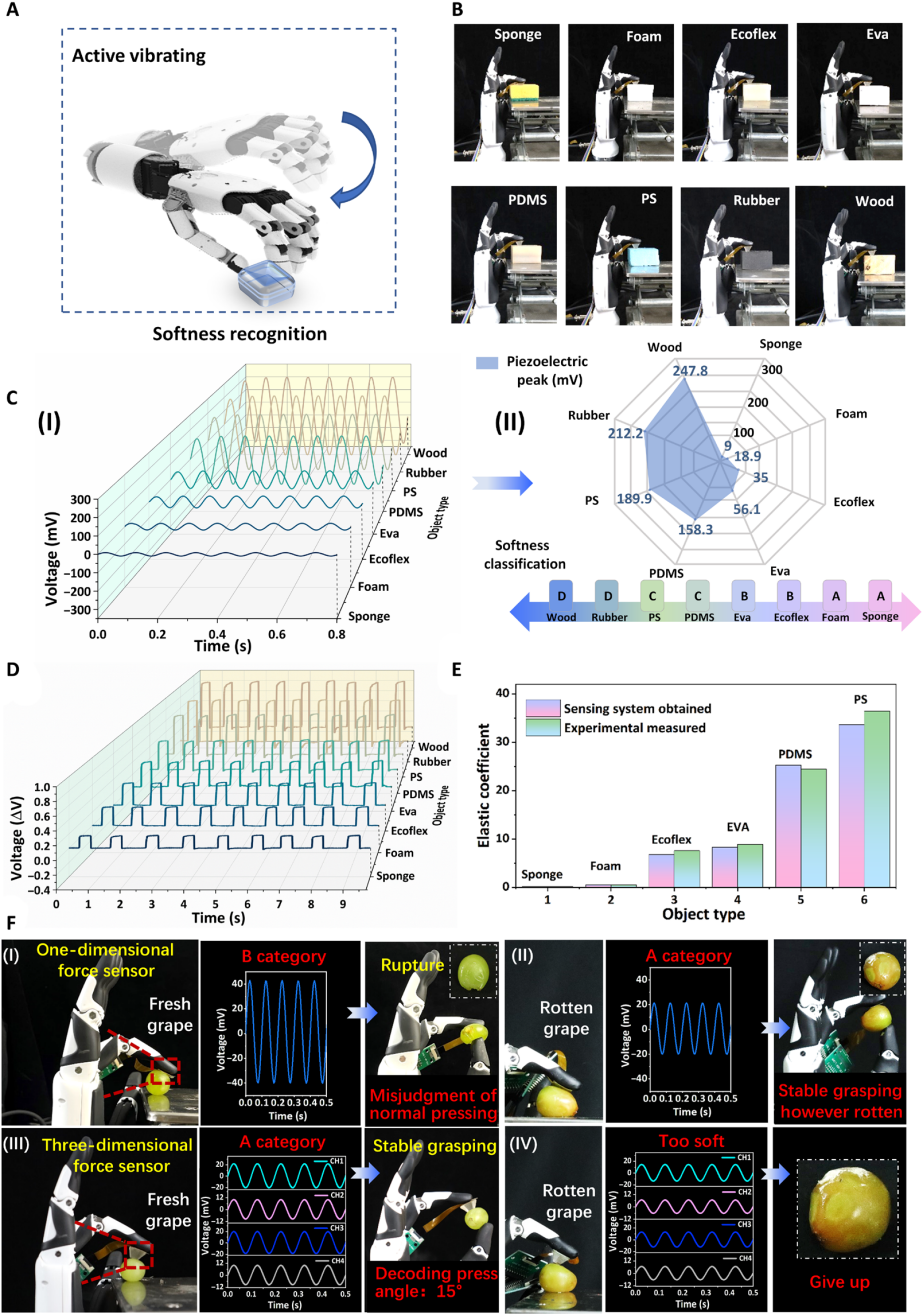
Softness recognition of the haptic sensor based on dynamic classification and static measurement strategies. (**A**) Schematic illustration of the softness recognition approach used by a robotic manipulator using active vibrating motion. (**B**) Photographs depicting a robotic manipulator touching eight different objects with varying degrees of softness, including sponge, foam, Ecoflex, PDMS, EVA, PS, rubber, and wood. (**C**) Real-time piezoelectric outputs and their corresponding voltage peaks obtained from the piezoelectric mode while touching the eight objects. (**D**) Relative voltage changes in the piezoresistive mode when the robotic manipulator applies pressures of 0.5, 2, 5, and 10 N onto the eight objects. (**E**) Comparison of the elastic coefficients of the measured objects between theoretical predictions and experimental demonstrations. (**F**) Comparative experiment involving grape grasping by a robotic manipulator based on (I and III) one-dimensional force and (II and IV) three-dimensional force sensing feedback. It shows that, with the one-dimensional force feedback, fresh and rotten grapes are damaged and stably grasped by the robotic arm due to misjudgment of softness. With the decoupled force feedback from the three-dimensional haptic sensor, the fresh grape is stably grasped without damage, while the rotten grape is given up for grasping.

As the haptic sensor comes into contact with an object in the form of active vibration (Fig. 3A), the dynamic response of the piezoelectric layer is designed to preliminary evaluate the softness. To further validate the ability of the sensing system to discriminate softness, we mounted the sensor on a robotic manipulator and recorded dynamic changes in electrical output. The robotic manipulator touches eight standard samples [sponge, foam, Ecoflex, polydimethylsiloxane (PDMS), ethylene vinyl acetate (EVA), polystyrene (PS), rubber, and wood (Fig. 3B)] with a force of 1 N at a vibration frequency of 10 Hz. The measured results demonstrate how the piezoelectric voltage changes when the sensor interacts with various sample materials (Fig. 3C, I). The sponge has a comparatively low elastic modulus, which reduces the deformation rate and results in a weaker piezoelectric signal of 9 mV originating from the dynamic response characteristics of the loading rate. Real-time curves of the sensor reveal a stable piezoelectric output across seven cycles when exposed to samples exhibiting different levels of softness. Consequently, the actively vibrating piezoelectric layer provides voltage feedback with varying peaks based on the dynamic response characteristics (Fig. 3C, II), thus presenting an alternative method to conventional quasistatic measurements for the preliminary classification (e.g., A, B, C, and D) of object softness.

The synergistic effect of piezoelectric and piezoresistive modules further allows for the deconvolution of the dynamic and static stimuli during the complex identifying processes. Once the object’s softness was preliminarily classified, the robotic manipulator would apply a corresponding contact force based on the classification results, allowing the piezoresistive layer to yield different feedback (Fig. 3D). The measuring mechanism is based on contact mechanics theory, which takes into account the elastic deformation of the object due to contact forces and evaluates the essential softness performance through the elastic coefficient (text S2). The quantitative testing results show good agreement with the results obtained from experimental observations, with an average relative error controlled within 10% (Fig. 3E and table S2). Thus, the designed measuring strategy uses the synergistic effect of piezoelectric and piezoresistive modules to decode the softness, providing a nondestructive methodology for quantitative identification.

To improve the accessibility of this softness recognition approach, we further validated the measurement ability of sensors and objects at an arbitrary interaction angle. In the application scenario of intelligent robots, the practical task of gripping objects of different shapes and sizes is arbitrary for robots and cannot guarantee normal orientation, resulting in complexity when applying pressing force that involves both normal and shear forces. As a proof-of-concept demonstration, when a grape of unknown softness is arbitrarily pressed, the one-dimensional sensory feedback only classifies the softness of the grape as category B based on a single-channel piezoelectric voltage. The FEA results indicate that this phenomenon occurs due to the presence of additional shear strain in addition to the normal strain when the sensor comes into oblique contact with the grape, resulting in a higher voltage output compared to when it is pressed normally against the surface (fig. S14). Consequently, the robotic manipulator applies a gripping force of 2 N according to the classification, resulting in the rupture of the fresh grape (Fig. 3F, I). In contrast to the single information–based softness analysis strategy, the three-dimensional force sensor can decode the pressing angle (i.e., 15°) and make additional corrections, thus accurately classifying the softness and steadily grasping the fresh grape with a gripping force of 0.5 N (Fig. 3F, III). Meanwhile, a one-dimensional force sensor, used to detect grapes if these grapes have reached a state of deterioration, erroneously identified them as good due to recognition errors, enabling stable grasping (Fig. 3F, II). On the contrary, the three-dimensional force sensor accurately decodes the touch angle and identifies grapes that are rotten, thus avoiding their harvest and facilitating the efficient collection of only the suitable ones (Fig. 3F, IV). These experiments demonstrate that the proposed strategy can be regarded as a nondestructive, quantitative, and universal softness measurement approach for robots to perform delicate operations.

### Texture recognition approach assisted by spectrum analysis and deep learning

**Fig. 4.**
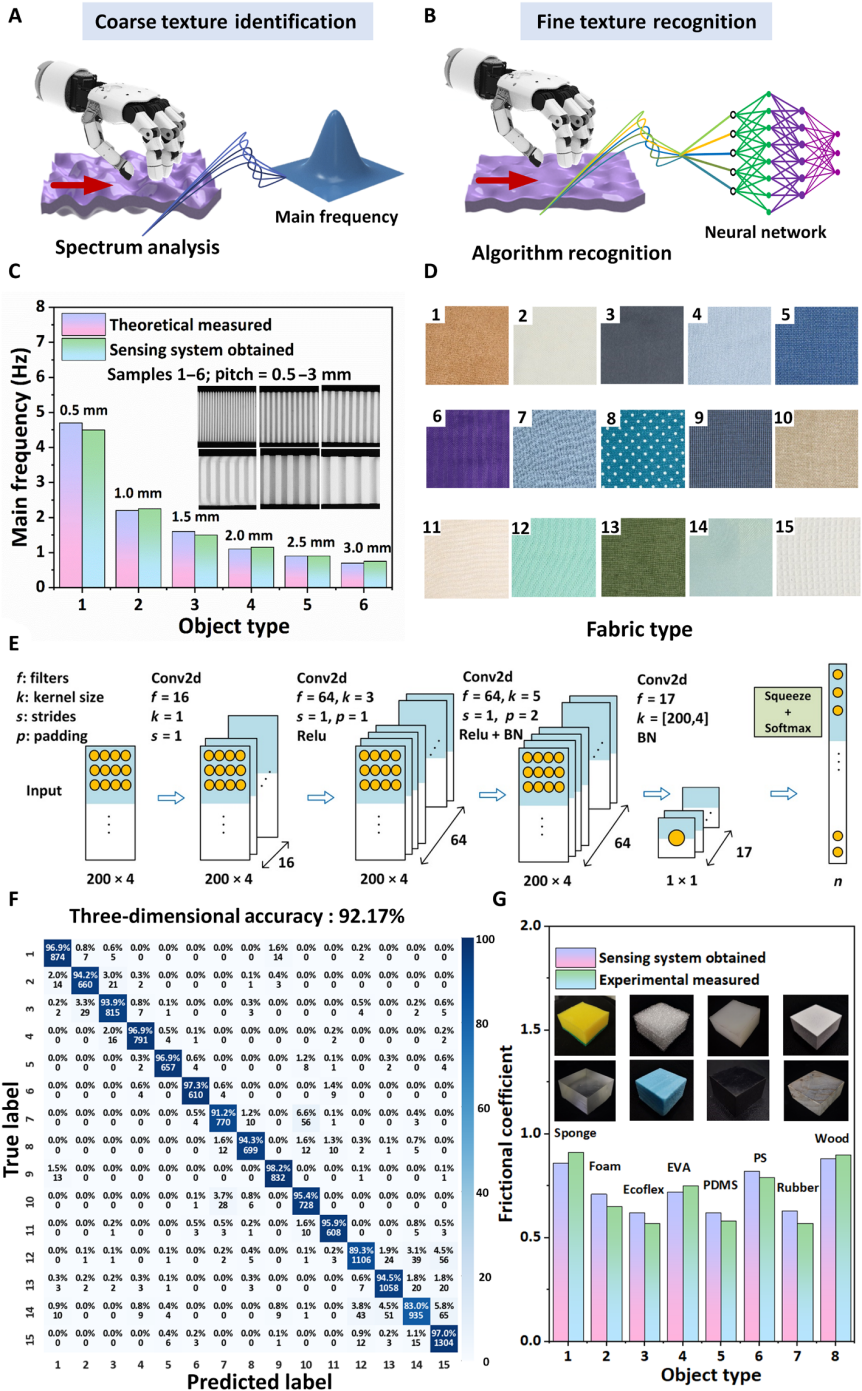
Texture discrimination of the haptic sensor assisted with spectrum analysis and deep learning. Schematic illustrations depict the identification of (**A**) coarse textures through spectral analysis, while (**B**) fine textures are recognized using neural network models. (**C**) Comparison of main frequency from six 3D-printed samples with spacings ranging from 0.5 to 3 mm between theoretical analysis and experimental demonstrations. (**D**) Optical images of 15 fabrics with diverse complex patterns. (**E**) Schematics of the process and parameters used in constructing the artificial neural networks. Rectified linear unit (Relu) followed by Batch Normalization (BN). (**F**) Confusion matrix for machine learning results on 15 fabrics using four channels of feedback. The color bar represents normalized accuracy. (**G**) Comparison of the frictional coefficients from touching eight different objects with varying degrees of texture, including sponge, foam, Ecoflex, PDMS, EVA, PS, rubber, and wood.

Tactile texture recognition is a complex process mediated by the spatial encoding of the geometrical properties of coarse textures and the vibrotactile encoding of fine textures. The haptic sensor was mounted on the robotic finger to perform sliding action, and the spectrum analysis on the acquired sensing data was first carried out to assist in identifying coarse textures (Fig. 4A). To evaluate the effectiveness of the haptic sensor in detecting coarse textures, we selected six 3D-printed samples with varying degrees of standard roughness for analysis. Upon observation, the materials’ surfaces exhibit micromorphological features characterized by peaks and dips, with two pitch widths along the sliding direction ranging from 0.5 to 3 mm (inset of Fig. 4C). The acquired sliding data are then subjected to a fast Fourier transform (FFT) to derive the frequency response (fig. S15). Results obtained from the FFT spectral analysis (Fig. 4C) reveal a decrease in the main frequency as the sample spacing increased, corroborating the theoretical calculation (see text S3 for details). This spectrum analysis method effectively detects periodic information in regular roughness patterns of surface textures, constrained by irregular fine textures, enabling comparisons between observed and theoretically predicted frequencies. Compared to commercial probe-based texture analysis strategies, flexible haptic sensing is more convenient and economical and does not damage the sample surface, thus showing potential for applications in future intelligent robotics and industrial manufacturing.

Recognizing fine textures presents a notable challenge for sensors as these surfaces exhibit irregular patterns that include aperiodic variations in amplitude and spatial intervals. Accurately quantifying textured surfaces requires the identification of features mediated by the transduction and processing of vibrations generated during scanning. To address this challenge, emerging deep learning algorithms can effectively extract complex information from elaborated feedback and, although they rely on a large amount of data, still have the potential to assist in identifying fine and irregular textures (Fig. 4B). Fabrics with delicate and intricate surface textures were chosen as suitable candidates for testing samples (Fig. 4D). A convolutional neural network (CNN) was developed to effectively extract features from the original complex signals (Fig. 4E). The confusion map demonstrates that the CNN method, using one-dimensional information, enabled the manipulator to achieve a texture recognition accuracy above 54.55% (fig. S16). Notably, an improvement in recognition accuracy to 92.17% was achieved by incorporating features learned from three-dimensional feedback (Fig. 4F). By integrating haptic feedback from different dimensions, a more comprehensive set of features related to slip adhesion and geometric deformation from the measured objects could be obtained, thus effectively enhancing recognition accuracy. The demonstration indicates that the haptic sensor is capable of detecting various textures, including coarse and fine patterns, by simultaneously detecting static and dynamic stimuli.

To further improve the applicability of this haptic sensor in robotic manipulator grasping, the quantitative analysis in the texture recognition process is enlightening for acquiring abundant information related to sliding friction. The constant normal pressure is performed using the feedback of the piezoresistive module, while the dynamic shear force is determined using the piezoelectric module. By decoupling the signal from the flexible three-dimensional force sensor in the piezoelectric and piezoresistive mechanism, the friction coefficient associated with the minimum gripping force required for the measured objects can be obtained. To validate the accuracy, these values are compared to actual friction coefficients measured using a frictional experiment regarding various objects (Fig. 4G and table S3). The measurement error is found to be within a range of 10%, indicating satisfactory performance of the sensor in measuring friction coefficients. This demonstration shows the ability of this sensor to accurately determine the surface friction coefficient of objects through the synergistic effect of the piezoelectric and piezoresistive modules, providing an underlying basis for the quantification of texture perception.

### Medical application of the bimodal haptic sensors on clinical feature identification

**Fig. 5.**
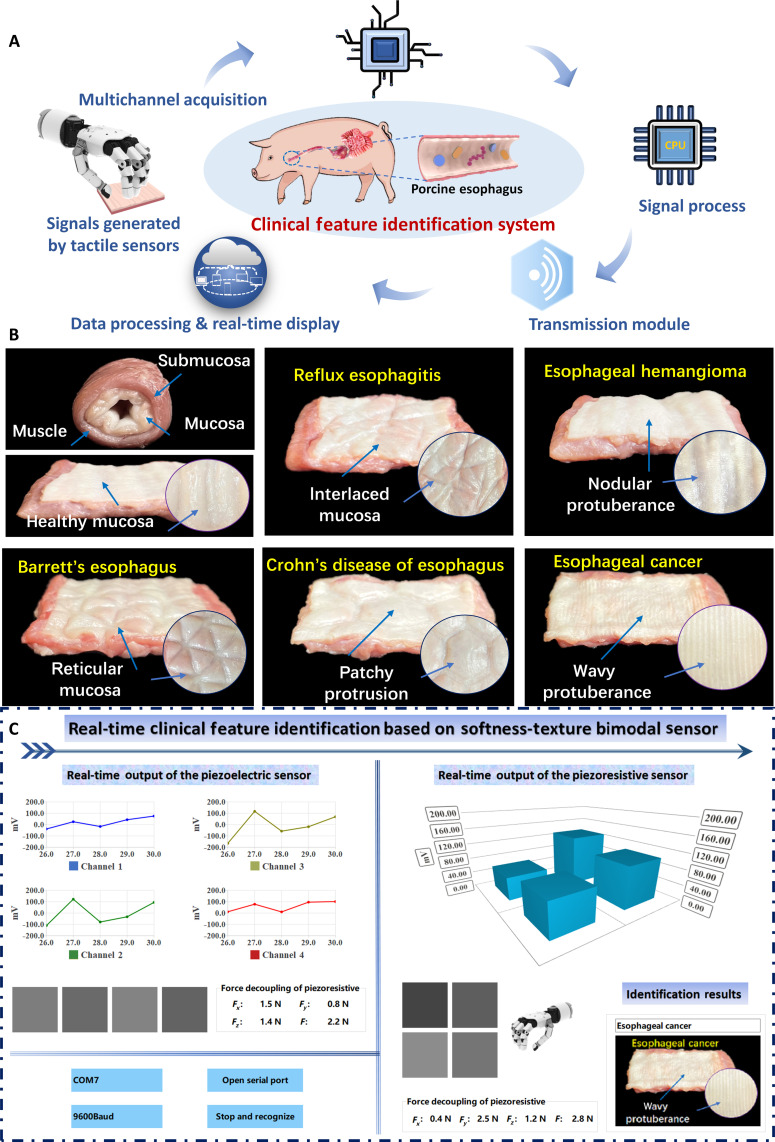
Medical application of clinical feature identification by the bimodal haptic sensor and the interactive interface. (**A**) Schematic illustration of the clinical feature identification system, containing the signal output information of haptic sensor, multichannel acquisition, signal processing on central processing unit (CPU), wireless transmission, and real-time display modules. (**B**) Optical images of porcine esophagus with diverse textures and softness, associated with different pathological features (i.e., reflux esophagitis, esophageal hemangioma, Barrett’s esophagus, Crohn’s disease of esophagus, and esophageal cancer). (**C**) Real-time presentation of the computer interface showcasing the results of clinical feature recognition.

The haptic sensor, capable of identifying softness and texture, is expected to enable clinical feature identification. The wrinkling morphology and elastic modulus of the esophageal mucosa are believed to have a crucial physiological role in maintaining the health of biological tissues, which are used to evaluate overall health status and identify diseases. To demonstrate the practical feasibility of the haptic sensor in identifying clinical features based on softness and texture recognition, a smart robotic manipulator is equipped with integrated bimodal sensing modules, a data acquisition and transmission module, and a display module (Fig. 5A). The multichannel acquisition module could accurately record signals containing detailed characteristics of esophageal mucosa. The signals were then transmitted to the computer for machine learning, and the results were displayed on the computer display interface.

The surface of the esophageal mucosa exhibits various patterns of wrinkling or nonuniformity. The associated elastic modulus can be affected by diseases induced by inhomogeneous growth, such as reflux esophagitis, esophageal hemangioma, Barrett’s esophagus, Crohn’s disease of esophagus, and esophageal cancer (*68*–*72*). To obtain sufficient esophageal samples for recognition, the simulated morphologies of the unhealthy porcine esophagus were used as a model system because of its ease of availability and similarity to the human esophagus. For the purpose of simulating real pathological phenomena with diverse morphologies, we applied mechanical stimulation on the surface of the porcine esophagus to create particular characteristics resembling various clinical conditions (Fig. 5B). The recognition process was executed to ensure controllable and repeatable sliding, and these robotic sliding actions were repeated 50 times. The obtained data of each status were then used for training (80%) and testing (20%) and labeled according to classes. After training by the CNN (fig. S17), the accuracy for clinical feature identification reached 98.44% (fig. S18).

To explore the real-time clinical feature identification ability of our bimodal sensor, the robotic manipulator continuously touched the porcine esophagi, and the final prediction results with the assistance of a pretrained machine learning model were intuitively displayed on the software display interface (Fig. 5C and movie S1). By relying on sensing feedback from softness and texture recognition, the intelligent robotic manipulator can effectively recognize pathological features. This demonstration of the real-time palpation platform facilitates the precise identification of esophageal characteristics of inflammation or other lesions, which are crucial for accurate disease staging and treatment planning. Real-time testing on porcine esophageal tissues has demonstrated the sensors’ relevance in clinical settings, effectively differentiating between normal and diseased tissues. Collectively, these findings clearly indicate that sensors capable of detecting the physical properties of softness and texture are unique tools in the field of gastroenterology, where these devices can provide valuable data for diagnosis, treatment tracking, and disease monitoring.

### Agricultural application of the bimodal haptic sensors on intelligent picking

**Fig. 6.**
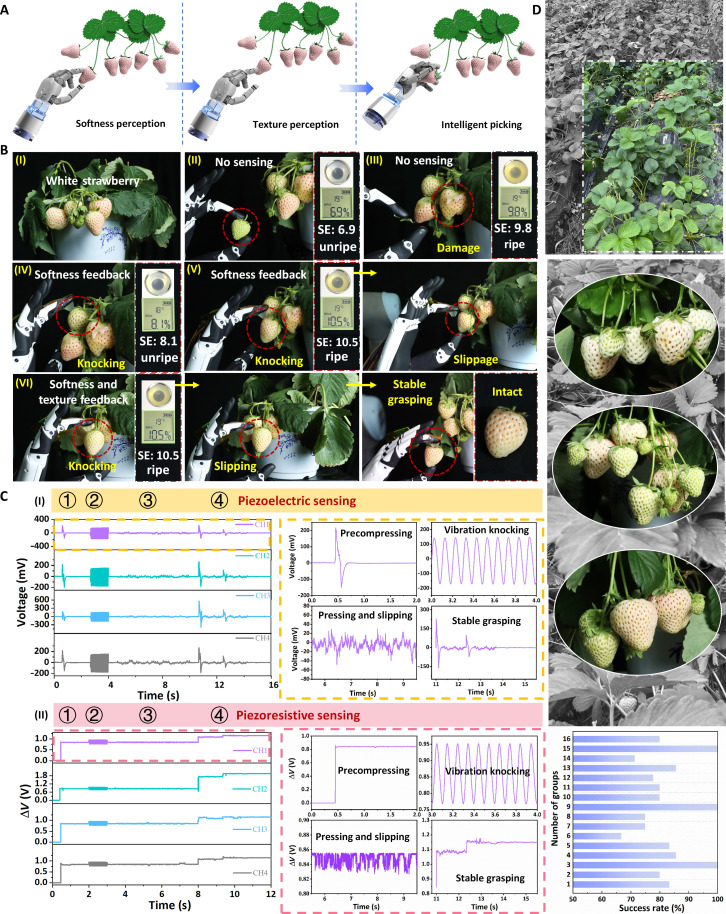
Agricultural application of the bimodal haptic sensor on intelligent picking white strawberries. (**A**) Schematic illustration of a robotic manipulator integrated with the bimodal sensor for grasping white strawberries via the softness and texture perception. (**B**) Processes of the robotic manipulator grasping white strawberries with unknown maturity. (I) Optical image of potted white strawberries. Without the sensing feedback, the picked strawberries suffer from the problem of (II) unripe or (III) damage during grasping. An enlarged optical image reveals the validation of a sweetness meter to quantitatively measure the strawberry’s sweetness, represented by Sucrose Equivalent (SE). Equipped only with feedback functions for softness recognition, the robotic manipulator (IV) bypasses unripe strawberries and (V) carefully picks ripe ones within maximum force to avoid damage, but occasionally, its gentle grip causes the strawberries to slip. (VI) With softness and texture feedback, the robotic manipulator executes the prepressing, vibration knocking, and slipping motions to achieve stable grasping. (**C**) Measurements of piezoelectric and piezoresistive outputs, providing softness and texture feedback, were obtained from the bimodal sensor during the intelligent picking of white strawberries. (**D**) Success rate of recognizing and picking a large number of white strawberries with different shapes and sizes.

To address the challenges of steadily picking ripe white strawberries in the absence of visual maturity cues, we propose an innovative approach for in situ robotic sensing that focuses on the simultaneous sensing of softness and texture for determining the appropriate operating force and realizing intelligent picking (Fig. 6A). When picking strawberries, it is recommended to manipulate the portion close to the stem as studies have shown that the best picking point is generally 13 to 20 mm above the calyx along the fruit stem (*73*). Consequently, the robotic manipulator touches the upper portion of white strawberries to assess ripeness and ensure reliable grasping. To further accurately evaluate the ripeness of white strawberries, we used a professional-grade sweetness meter for quantification. For comparison, a grasping task using the robotic manipulator without integrating the haptic sensor was first implemented (movie S2). It shows that the picked strawberries are either underripe (Fig. 6B, II) or fully ripe but damaged (Fig. 6B, III), primarily because there is a lack of sensory feedback. In contrast, we then mounted a haptic sensor, equipped solely with softness recognition capabilities, on the robotic manipulator to demonstrate the task of grasping white strawberries of indeterminate ripeness (movie S3). The softness feedback from the recognition result prompts the robotic manipulator to bypass this unripe strawberry, and instead, it vibrates and knocks a different one (Fig. 6B, IV). After recognizing a mature strawberry, the robotic manipulator reduces its gripping force for the task, but the strawberry slips due to insufficient force to overcome the drag (Fig. 6B, V).

To further demonstrate the ability of our bimodal sensor with softness and texture sensing feedback in grasping challenges, the intelligent robot works by the following steps (Fig. 6B, VI, and movie S4): (i) The robotic manipulator performs a preload motion over the white strawberry, acquiring data through both piezoelectric, which generates an instantaneous signal upon contact, and piezoresistive sensors, the latter producing a continuous voltage response (Fig. 6C). (ii) The further knocking action implemented by the robotic manipulator induces periodic fluctuations in piezoelectric (peak value: 147 mV) and piezoresistive sensing signals, leading to the classification of the white strawberry’s softness as category C and determining a maximum gripping force of 5 N. (iii) Upon the softness recognition, the robotic manipulator executes a sliding motion with a slip velocity of 12.76 mm/s to discern its texture, where decoupling normal force through piezoresistive modules and shear force via piezoelectric modules enables an initial pressure determination of 2.16 N using a 0.72 friction coefficient (i.e., where *F*_initial_/μ ≤ 0.6 *F*_max_). (iv) During the gripping process, the rotation speed of the index finger on the robotic manipulator is 45°/s at the proximal end and 60°/s at the distal end. The robotic manipulator determines a shear force of 1.3 N by analyzing slip signal fluctuations from the piezoelectric sensor and decoupling from the piezoresistive sensor, noting that the self-locking angle falls outside the friction cone, prompting an adaptive increase in gripping force until the *F_x_*/*F_z_* ratio is safely within the friction cone. Sensing signals from both piezoelectric and piezoresistive modules indicate normal and shear forces of 2.37 and 1.66 N, respectively, facilitating stable robotic grasping.

Robotic adaptive grasping demonstrates the capability of this bimodal sensor to recognize softness and texture, thereby benefiting intelligent picking. To adapt our method to real-world scenarios, we collected and evaluated the recognition and picking processes of 101 white strawberries, varying in shape and size (Fig. 6D). The variation in success rates among the groups is primarily due to the inconsistent number of strawberries, which is influenced by natural growth conditions of each plant. The overall success rate was 82.18% (table S4), which is based on a total of 101 white strawberries with 83 successfully harvested samples, indicating high reliability for practical applications. These results collectively show that our bimodal haptic sensor is well equipped to perform high-skill tasks, which are essential for intelligent picking and help prevent losses that can occur with manual or indiscriminate mechanized harvesting in agriculture. On the basis of this proof of concept, the remaining challenges in practical applications will be addressed through further miniaturizing the bimodal multidimensional haptic sensor. We have developed a compact bimodal multidimensional sensor measuring 2.3 mm in upper side length, 5 mm in bottom side length, and 3.1 mm in height (fig. S19). This represents a 94% reduction in size compared to the original dimensions (upper and bottom side lengths of 13 and 6 mm, respectively; height of 7.5 mm). The performance investigations demonstrate that the sensor, despite its compact size, accurately detects three-dimensional forces with high sensitivity (fig. S20) and excellent stability (fig. S21). Multidimensional sensors of various sizes have broad potential applications and can be tailored to specific needs to ensure appropriate volumes. We believe that integrating the haptic sensor into moving components and mobile devices, such as prosthetics, robotic fingers, and minimally invasive surgery instruments, would greatly benefit many fields.

## DISCUSSION

In summary, this paper reports a methodology for simultaneously quantifying material softness and texture using a bimodal haptic sensor, enabling precise elastic and frictional coefficients measurements via the synergistic effect of three-dimensional static and dynamic stimuli feedback. The proposed softness recognition method, using piezoelectric predictive classification and piezoresistive quantitative detection, provides scarless assessment from various touch angles, overcoming the difficulties of damage and accuracy that are commonly encountered in standard static and dynamic softness measurements. The texture identification approach elucidates the complex evolution of normal and shear stimuli resulting from deformation friction and interfacial adhesion during texture recognition, thereby compensating for the common neglect of sliding friction force in current measurement methods. Accordingly, quantifying the physical parameters of softness and texture identification demonstrates a nondestructive, precise, and easy-to-use measurement technology, which is a universal method that allows for determining an appropriate grasping force to minimize damage and slippage of fragile objects. Real-time visualized recognition platform is further demonstrated while the robotic manipulator identifies healthy and pathological porcine mucosal features with 98.44% classification accuracy, achieving objective and reliable palpation of living tissues to effectively monitor and diagnose health disorders. The softness and texture sensing feedback provided by this haptic sensor endow the robotic manipulator with human-comparable behavior to accomplish challenging tasks of adaptively grasping mature white strawberries and achieving intelligent picking, thereby making it one of the most pragmatic and effective designs to be a promising option for various applications. We anticipate that our haptic sensor design has the potential to facilitate the attainment of multimodal haptic perception in various areas of robotics, particularly in tasks involving stable grasping, digitized health monitoring, and haptic interfaces for advanced humanoid robots.

## MATERIALS AND METHODS

### Preparation of the flexible piezoelectric film

The P(VDF-TrFE) powder (Piezotech) was mixed with *N*,*N*-dimethylformamide in a molar ratio of 70:30. Stirring the mixture at room temperature for 2 hours resulted in a uniform solution. The solution was then cast onto a petri dish and annealed at 120°C for 2 hours to enhance crystallinity, forming the P(VDF-TrFE) film. After peeling the film off from the petri dish, it was polarized by placing it in a silicone oil bath with an electric field of 70 megavolts m^−1^ for 30 min. This polarization process led to a high piezoelectric property in the film, such as a piezoelectric strain constant *d*_33_ value of 28 pC N^−1^ along the z-direction. The *d*_33_ values of piezoelectric films were measured by the quasistatic *d*_33_ tester (ZJ-6A of the Chinese Academy of Sciences) at room temperature.

### Preparation of the flexible piezoresistive film

For the initial synthesis, multiwalled carbon nanotubes (Aladdin Tech. Inc.), sodium dodecylbenzene sulfonate surfactant, SiO_2_ nanoparticles, and the silane coupling agent KH-560 were combined with cyclohexane in a weight ratio of 5:10:3:2:1000. The mixture was mechanically stirred at 90°C for 20 min to achieve a uniform solution. Subsequently, GD401 silicone rubber was added to the cyclohexane solution to form a hybrid mixture, which was vigorously stirred for 30 min. This mixture was then combined in a beaker and sonicated for 30 min, followed by mechanical stirring for 1 hour to ensure uniform dispersion. After the mixed suspension transformed into a viscous solution, it was transferred to a vacuum drying oven for 10 min to eliminate air bubbles.

### Fabrication of the bimodal three-dimensional force sensor

Gold electrodes (150 nm in thickness) were evaporated onto a 100-μm-thick PI film using an electron beam evaporator (DZS-500) to create the distributed electrodes. The piezoelectric layer (thickness: 28 μm) and piezoresistive layer (thickness: 102 μm) were then assembled layer by layer to form a dual-mode sensor. The entire bimodal pressure sensor was encapsulated using 50 μm thick polyethylene terephthalate (PET) films on both sides. After cleaning the 3D-printed template with deionized water, a square frustum-shaped PDMS bump (upper side length: 6 mm; bottom side length: 13 mm; height: 7 mm) was prepared by casting a mixture of PDMS (Sylgard 184, Dow Corning) with a ratio of part A to part B at 10:1 into the template. The surface treatment was applied to both the PET film and the PDMS bump using a surface treatment agent (Gubaili 707), followed by a uniform deposition of a commercially available dispersion (Jule J-808) onto the PET substrate. The PDMS bump was then placed at the top and cured at 40°C for 30 min to assemble the bimodal three-dimensional force sensor. Adhesion tests (fig. S22) revealed that three sensing devices consistently exhibited a maximum adhesive force (e.g., 32.2, 34.6, and 32.1 N), surpassing the force required for their intended application.

### FEA of the sensors

FEA was conducted using the commercial software COMSOL (version 6.2, standard) to study the deformation in sensing layers. The interface between the PI and PET layers was set with continuity conditions to simulate the bonding behavior. The bump structure, sensing modules, and encapsulation layers were modeled using free tetrahedral meshing solid elements. The mechanical properties of the materials were set as follows: PI and PET were modeled with linear elastic behavior, with an elastic modulus of *E*_PI_ = 7.9 GPa and a Poisson’s ratio of ν_PI_ = 0.36 for PI and an elastic modulus of *E*_PET_ = 6.9 GPa and a Poisson’s ratio of ν_PET_ = 0.43 for PET. An elastic modulus of *E*_PDMS_ = 750 kPa and a Poisson’s ratio of ν_PDMS_ = 0.49 were for PDMS, and an elastic modulus of *E*_Piezoresistive_ = 127 MPa and a Poisson’s ratio of ν_Piezoresistive_ = 0.24 were for the piezoresistive layer.

### Measurements of the sensing performance of the sensors

The voltage output of the piezoelectric sensor was recorded by a piezoelectric data acquisition system (Beijing Keshang Instrument Technology Co. Ltd). For high-frequency loading, the sensor was positioned under a cylindrical probe, operated by an exciter, and powered by a signal from a high-precision network data analyzer that was amplified by a power amplifier. The force magnitude input to the sensor was measured by a calibrated piezoelectric force transducer with a sensitivity of 10 mV N^−1^, and the output from this transducer was processed through a charge amplifier before recording on a high-precision network data analyzer. The electrical output of the piezoresistive sensor was measured through a semiconductor parameter analyzer (4200-SCS, Keithley). The analyzer was configured as a current supply, delivering a bias current to the sensor. This setup enabled the measurement of voltage changes resulting from the inherent resistance variation in piezoresistive sensors. Compression and tensile tests were conducted using a mechanical testing system (Legend2345, Instron).
