# Supplementary material for: Quantitative softness and texture bimodal haptic sensors for robotic clinical feature identification and intelligent picking
Source: Sci Adv. 2024 Jul 24;10(30):eadp0348. doi: 10.1126/sciadv.adp0348 (PMC11268415; doi:10.1126/sciadv.adp0348)
Supplement: Supplementary file 1 — Supplementary Text Figs. S1 to S22 Tables S1 to S4 Legends for movies S1 to S4 [file sciadv.adp0348_sm.pdf]

Science Advances
AAAS

Supplementary Materials for

Quantitative softness and texture bimodal haptic sensors for robotic clinical feature identification and intelligent picking

Ye Qiu *et al.*

Corresponding author: Huaping Wu, wuhuaping@gmail.com; Hanqing Jiang, hanqing.jiang@westlake.edu.cn

Sci. Adv. **10**, eadp0348 (2024)
DOI: 10.1126/sciadv.adp0348

The PDF file includes:

Supplementary Text
Figs. S1 to S22
Tables S1 to S4
Legends for movies S1 to S4

Other Supplementary Material for this manuscript includes the following:

Movies S1 to S4

Supplementary Text

Text S1. Theoretical analysis and sensing mechanism of three-dimensional forces perception

To elucidate the mechanism and theoretical basis of three-dimensional force perception, a decoupling analysis of three-dimensional force signals is conducted using a piezoelectric module as a representative example. It should be pointed out that assuming a piezoelectric sensor has a linear relationship with the applied force, the conversion coefficient is η_4. When the piezoelectric sensor is exposed to a normal force in the *z*-direction, its four dispersed sensing units react equally, resulting in similar changes in voltage across V_1 to V_4. This response is characterized by the transfer ratio η_1 and conversion coefficient η_4, where $V_i = \eta_1 \eta_4 F_z$ for i=1-4. For shear forces in the *x*-direction, V_1 (V_3) and V_2 (V_4) exhibit equal but opposite voltage responses, as determined by the coefficient η_2. Similarly, the voltage response ratio η_3 is associated with shear forces in the *y*-direction. Therefore, the relationship between the output voltage and the applied force can be expressed as:

$$\begin{bmatrix} V_1 \\ V_2 \\ V_3 \\ V_4 \end{bmatrix} = \begin{bmatrix} \eta_1\eta_4 \\ \eta_1\eta_4 \\ \eta_1\eta_4 \\ \eta_1\eta_4 \end{bmatrix} F_z \quad \begin{bmatrix} V_1 \\ V_2 \\ V_3 \\ V_4 \end{bmatrix} = \begin{bmatrix} \eta_2\eta_4 \\ -\eta_2\eta_4 \\ \eta_2\eta_4 \\ -\eta_2\eta_4 \end{bmatrix} F_x \quad \begin{bmatrix} V_1 \\ V_2 \\ V_3 \\ V_4 \end{bmatrix} = \begin{bmatrix} -\eta_3\eta_4 \\ -\eta_3\eta_4 \\ \eta_3\eta_4 \\ \eta_3\eta_4 \end{bmatrix} F_y \tag{S1}$$

The principle of superposition enables the determination of the voltage response for each piezoelectric module under three-dimensional forces, as detailed in equation (S1). Using this approach, the responses in channels V_1 to V_4 effectively decouple the three-dimensional force experienced by the sensor, as demonstrated in equations (S2) and (S3):

$$\begin{bmatrix} V_1 \\ V_2 \\ V_3 \\ V_4 \end{bmatrix} = \begin{bmatrix} \eta_1\eta_4 \\ \eta_1\eta_4 \\ \eta_1\eta_4 \\ \eta_1\eta_4 \end{bmatrix} F_z + \begin{bmatrix} \eta_2\eta_4 \\ -\eta_2\eta_4 \\ \eta_2\eta_4 \\ -\eta_2\eta_4 \end{bmatrix} F_x + \begin{bmatrix} -\eta_3\eta_4 \\ -\eta_3\eta_4 \\ \eta_3\eta_4 \\ \eta_3\eta_4 \end{bmatrix} F_y = \begin{bmatrix} \eta_1\eta_4 F_z + \eta_2\eta_4 F_x - \eta_3\eta_4 F_y \\ \eta_1\eta_4 F_z - \eta_2\eta_4 F_x - \eta_3\eta_4 F_y \\ \eta_1\eta_4 F_z + \eta_2\eta_4 F_x + \eta_3\eta_4 F_y \\ \eta_1\eta_4 F_z - \eta_2\eta_4 F_x + \eta_3\eta_4 F_y \end{bmatrix} \tag{S2}$$

$$\begin{cases} F_x = \dfrac{1}{4\eta_2\eta_4}(V_1 - V_2 + V_3 - V_4) \\ F_y = \dfrac{1}{4\eta_3\eta_4}(V_3 - V_1 + V_4 - V_2) \\ F_z = \dfrac{1}{4\eta_1\eta_4}(V_1 + V_2 + V_3 + V_4) \end{cases} \tag{S3}$$

Meanwhile, the output voltages along the x, y, and z directions will change with the applied normal and shear forces, which can be calculated by taking the average outputs of the four units as follows:

$$\begin{cases} V_x = \dfrac{V_1 - V_2 + V_3 - V_4}{2} \\ V_y = \dfrac{V_3 - V_1 + V_4 - V_2}{2} \\ V_z = \dfrac{V_1 + V_2 + V_3 + V_4}{4} \end{cases} \tag{S4}$$

The decoupled three-dimensional force remains closely related to the voltage output in both the normal and shear directions, as well as to the sensor's sensitivity, which can be expressed as follows:

$$\begin{cases} F_x = \dfrac{1}{k_x} V_x \\ F_y = \dfrac{1}{k_y} V_y \\ F_z = \dfrac{1}{k_z} V_z \end{cases} \tag{S5}$$

According to equations (S3-S5), the sensitivity along the x, y, and z directions can be obtained to further reveal the sensing mechanism of three-dimensional forces perception:

$$\begin{cases} k_x = 2\eta_2\eta_4 \\ k_y = 2\eta_3\eta_4 \\ k_z = \eta_1\eta_4 \end{cases} \tag{S6}$$

The relationship between the normal and force components F_x - F_z and converted component V_x - V_z, which is also called the calibration coefficients k_x - k_z, can be determined by experimental measurements.

Text S2. Theoretical analysis of softness measurement

The measuring mechanism of the elastic coefficient is based on a simple model to consider the elastic deformations of the sensors and the measured object. When an external force F is applied, the overall deformation x of the system is the summation of the deformations of both the sensor x_1 integrated in the fingertip and the measured object x_2, i.e., $x = x_1 + x_2$. According to force-displacement curve, the elastic coefficient of the sensor k_1 and measured samples k_2 under the force F can be derived as $k_i = \Delta F / \Delta x_i, i = 1, 2$. Then the equivalent elastic coefficient of the whole system can be further expressed as $k = \Delta F / \Delta(x_1 + x_2) = k_1 k_2 / (k_1 + k_2)$. Therefore, the elastic coefficient of the measured samples satisfies $k_2 = k k_1 / (k_1 - k)$.

Text S3. Theoretical analysis of texture measurement

A theoretical analysis based on haptic perception is carried out to reveal the regular texture measurement mechanism. In this analysis, a robotic hand applies a 1 N force to an object with uniformly spaced textures and slides at 4.5 mm/s, generating frictional vibrations. When a piezoelectric sensor encounters successive textures, it produces a regular pulsed electrical signal characterized by its main frequency f. The variation in the time intervals between the peaks of these signals, represented as Δt, can be utilized to calibrate the texture spacing ΔP. Thus, a relationship between f, ΔP, and the sliding speed v of the robotic hand can be obtained as:

$$f = \frac{v}{\Delta P} \tag{S7}$$

As the sensor moves smoothly across a sample at a constant velocity, with neighboring tactile units positioned at a specific distance d. The piezoelectric signals produced upon encountering the initial and subsequent gate textures are recorded at times t_1 and t_2, allowing for recording the time interval Δt. Consequently, the velocity at which the sensor traverses the object's surface can be described as:

$$v = \frac{d}{\Delta t} = \frac{d}{t_2 - t_1} \tag{S8}$$

According to equations (S7-S8), a direct relationship between the texture spacing ΔP and the main frequency f can be established:

$$f\,\Delta t = \frac{d}{\Delta P} \qquad \Delta P = \frac{d}{f\,(t_2 - t_1)} \tag{S9}$$

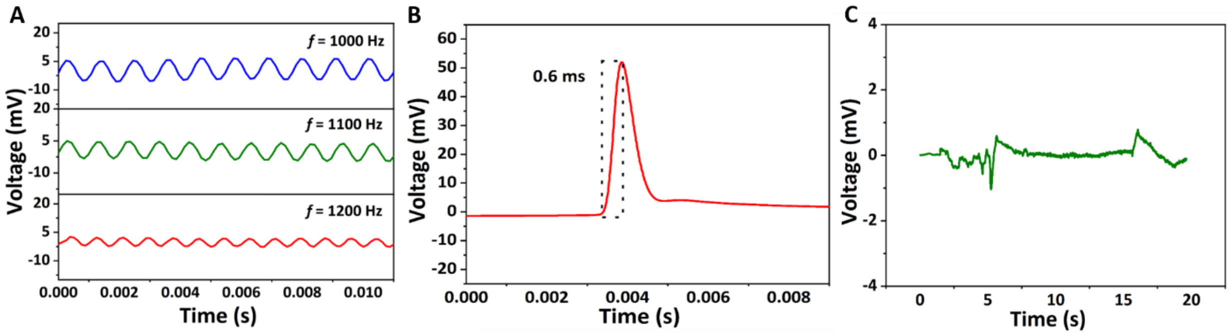

Fig. S1. The sensing performance of the piezoelectric layer. (A) Voltage responses of the piezoelectric layer at vibration frequencies of 1000, 1100, and 1200 Hz in the time domain. (B) Response time (i.e., 0.6 ms) of the piezoelectric layer. (C) Static pressure sensing performance of the piezoelectric layer for an applied force of 1 N.

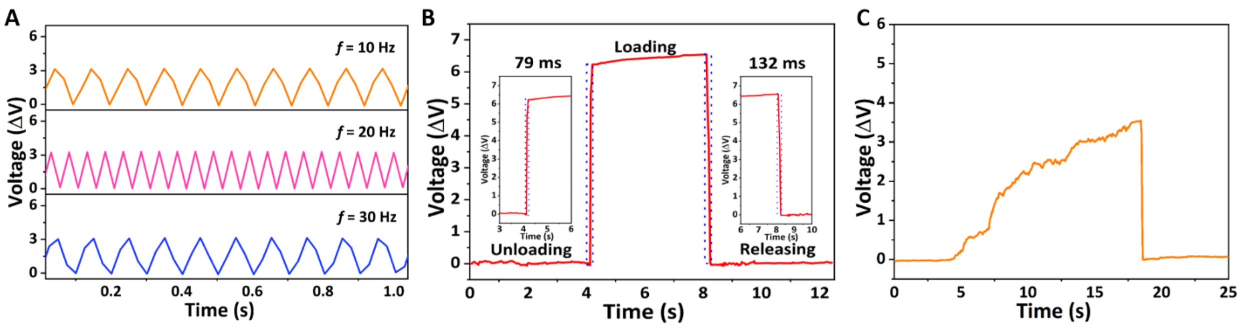

Fig. S2. The sensing performance of the piezoresistive layer. (A) Voltage changes of the piezoresistive layer at vibration frequencies of 10, 20, and 30 Hz in the time domain. (B) Response-relaxation time (i.e., 79 and 132 ms) of the piezoresistive layer. (C) Static pressure sensing performance of the piezoresistive layer for an applied force of 1 N with 0.5 mm/min loading rate.

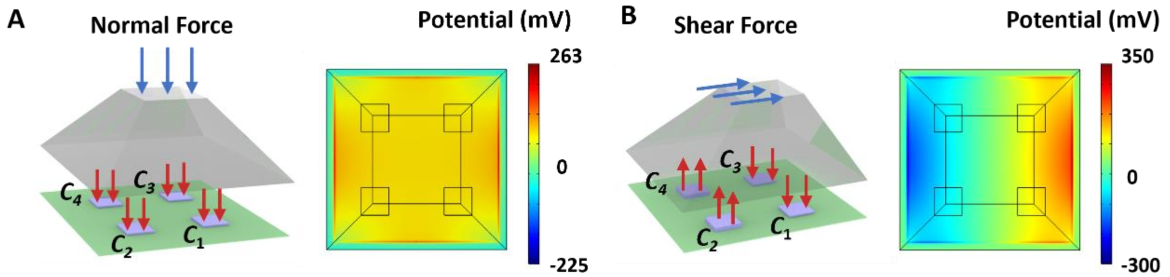

Fig. S3. Working principles and corresponding FEA predictions of multidimensional sensing implementation. The piezoelectric potential distribution of the sensing array under (A) normal and (B) shear forces of 2 N.

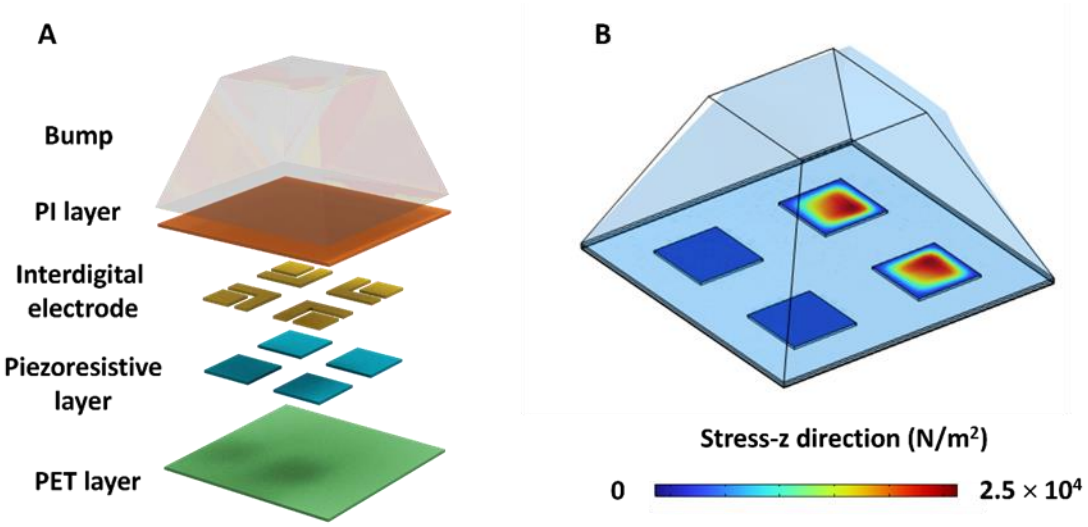

Fig. S4. The stress distribution of piezoresistive modules under the shear force. (A) The structural design of the piezoresistive module. (B) The stress distribution of piezoresistive sensing elements.

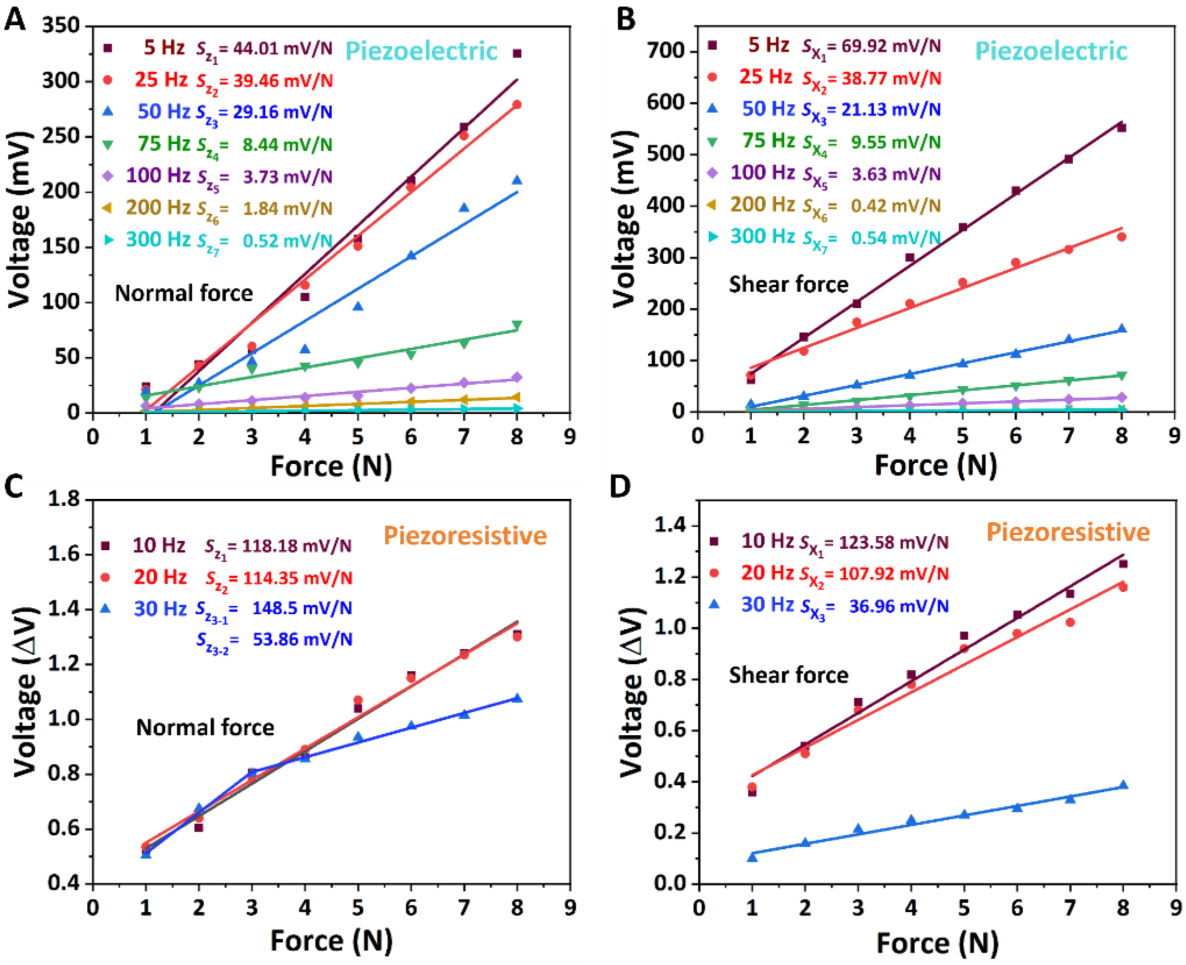

Fig. S5. The sensitivities of the bimodal sensor. The performance outputs of piezoelectric (A, B) and piezoresistive modules (C, D) were measured under applied normal and shear forces at frequencies ranging from 5 to 300 Hz.

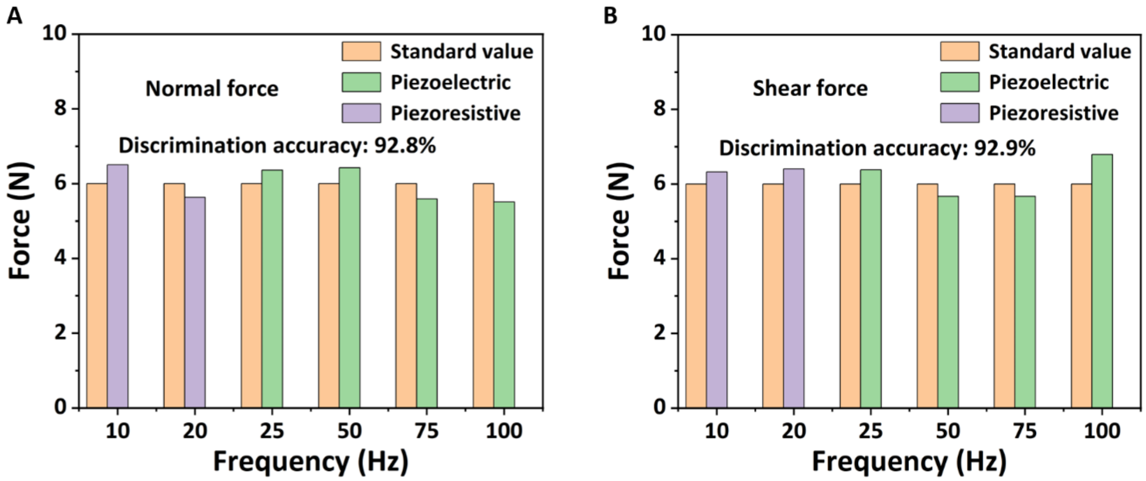

Fig. S6. The discrimination accuracy of the bimodal sensor. The comparison between the standard force value and the force measured by the sensor under (A) normal and (B) shear forces.

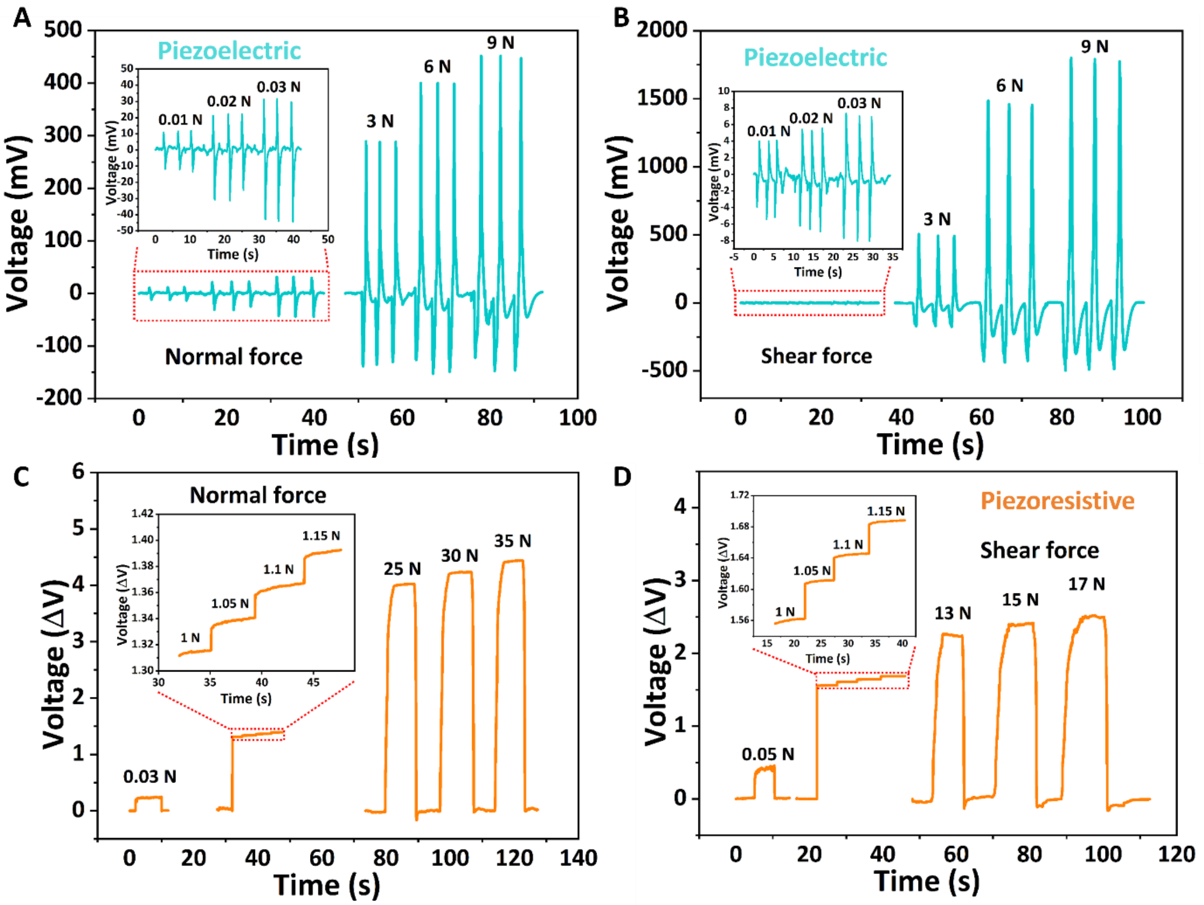

Fig. S7. The detection limit/range and pressure resolution of the bimodal sensor. The performance outputs of (A, B) piezoelectric and (C, D) piezoresistive modules were measured at the applied normal and shear forces.

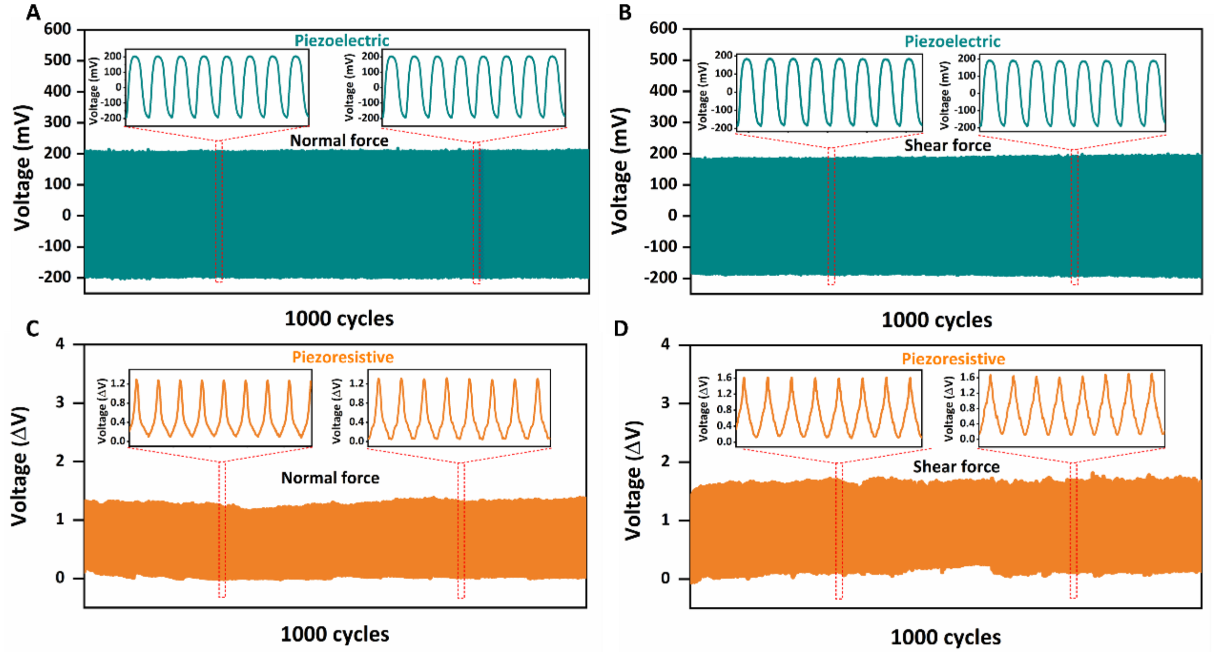

Fig. S8. The sensing stability of the bimodal sensor. Cycling tests of the (A, B) piezoelectric and (C, D) piezoresistive modules tested over 1000 cycles under normal and shear forces of 1 N.

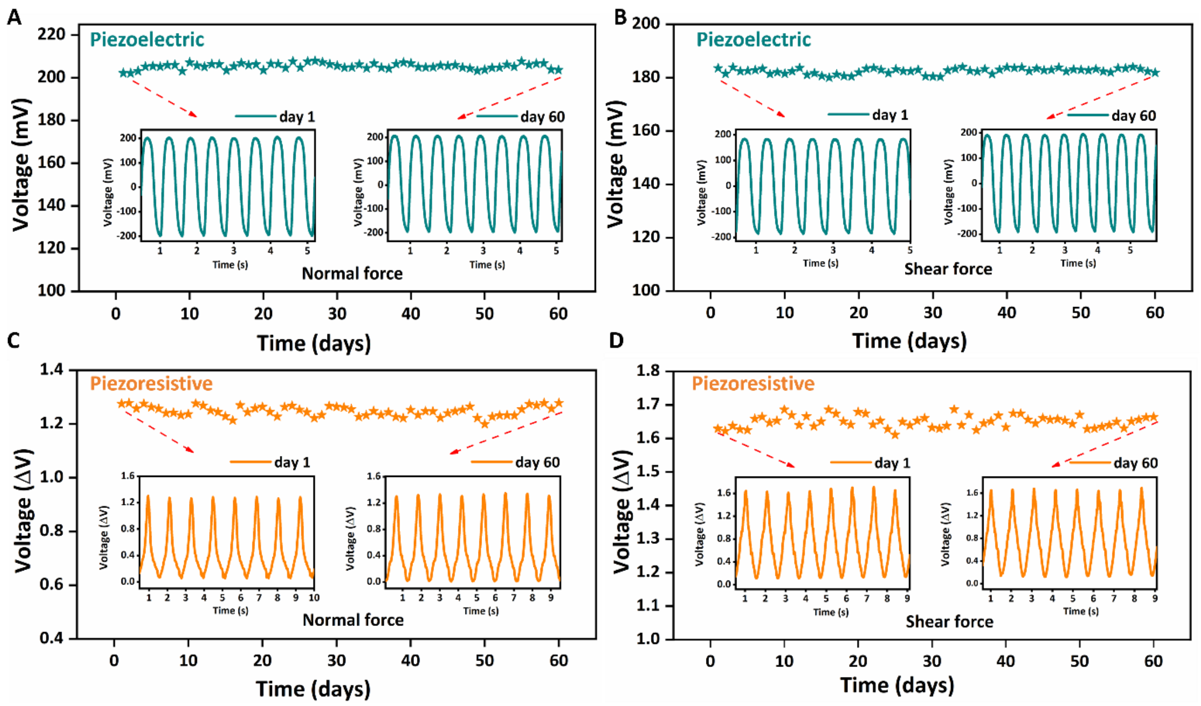

Fig. S9. The long-term stability of the bimodal sensor. The voltage output of the sensing modules based on (A, B) piezoelectric and (C, D) piezoresistive mechanisms upon normal and shear forces of 1 N, respectively.

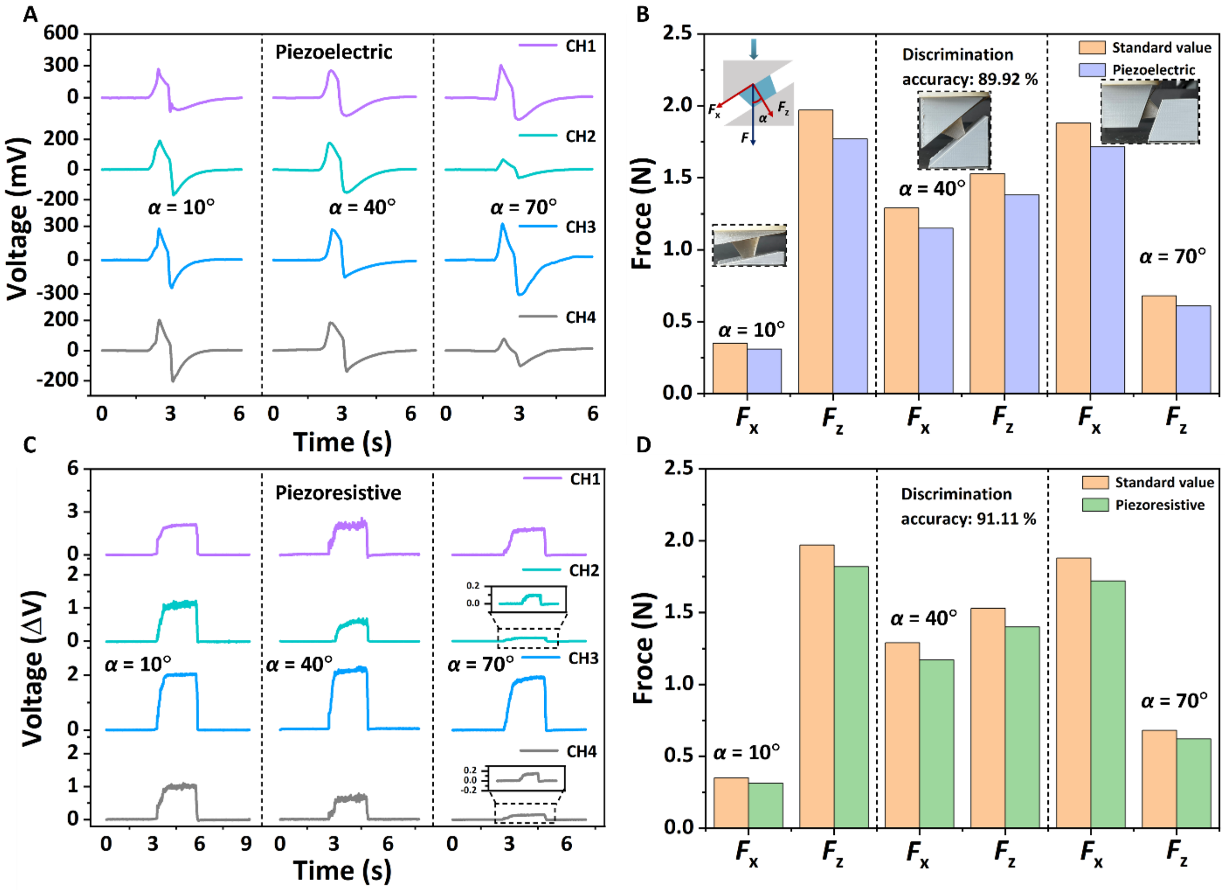

Fig. S10. The decoupling performance of the bimodal haptic sensor. The real-time output of the (A) piezoelectric and (C) piezoresistive modules. The decoupling accuracies of the (B) piezoelectric and (D) piezoresistive modules.

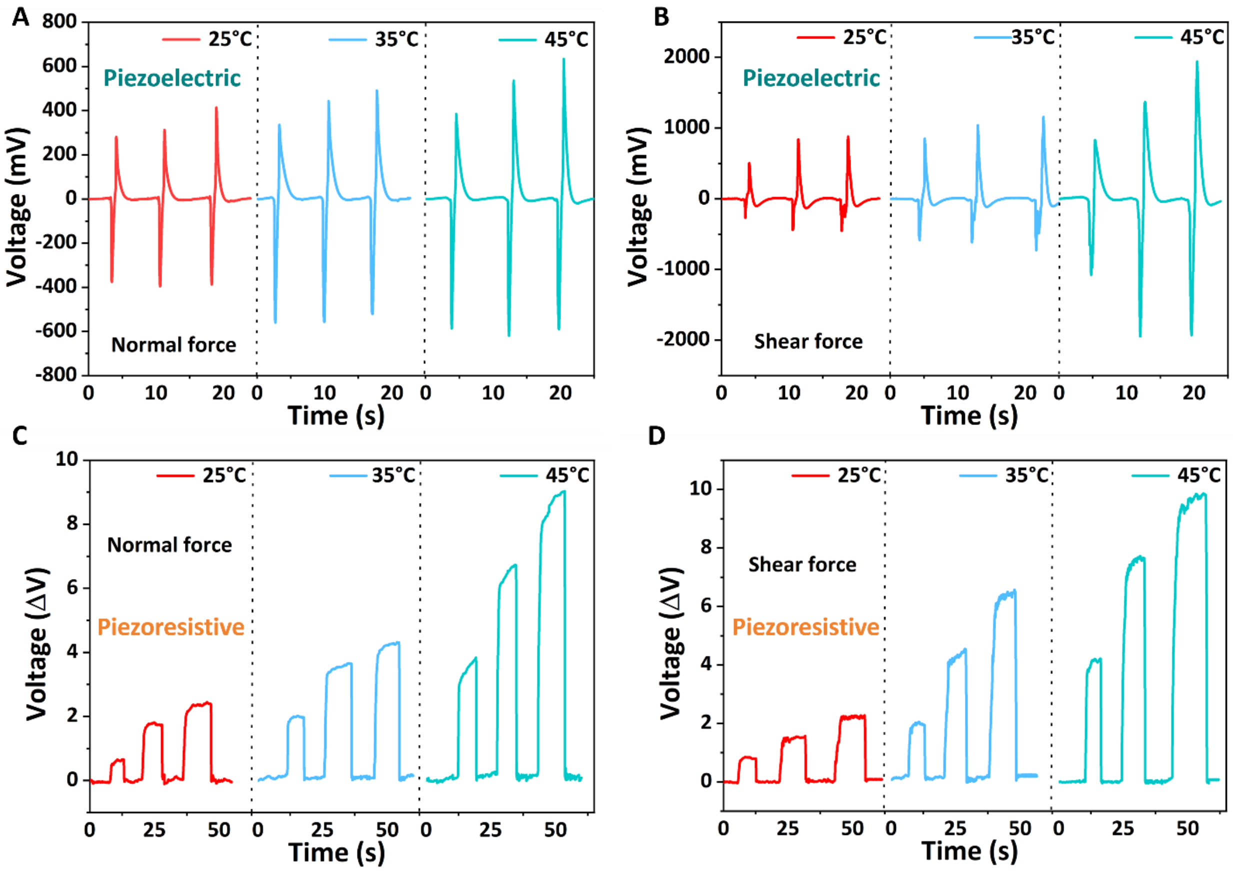

Fig. S11. The sensing performance of the bimodal sensor in response to variations in temperature changes. The performance outputs of (A, B) piezoelectric and (C, D) piezoresistive modules were measured at the applied force of 3, 4, 5 N and 1, 3, 5 N, respectively.

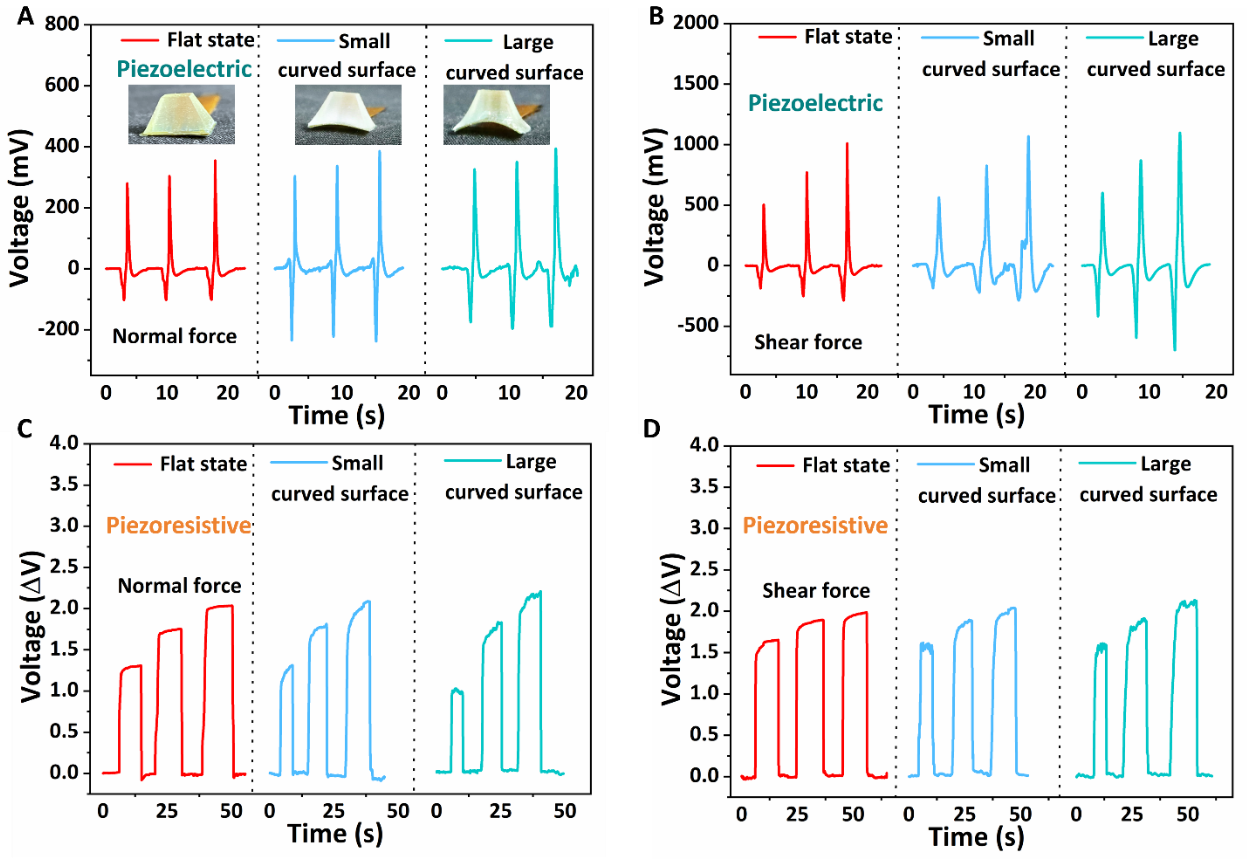

Fig. S12. The sensing performance of the bimodal sensor in response to variations in mechanical deformations. The performance outputs of (A, B) piezoelectric and (C, D) piezoresistive modules were measured at the applied force of 3, 4, 5 N and 1, 3, 5 N, respectively.

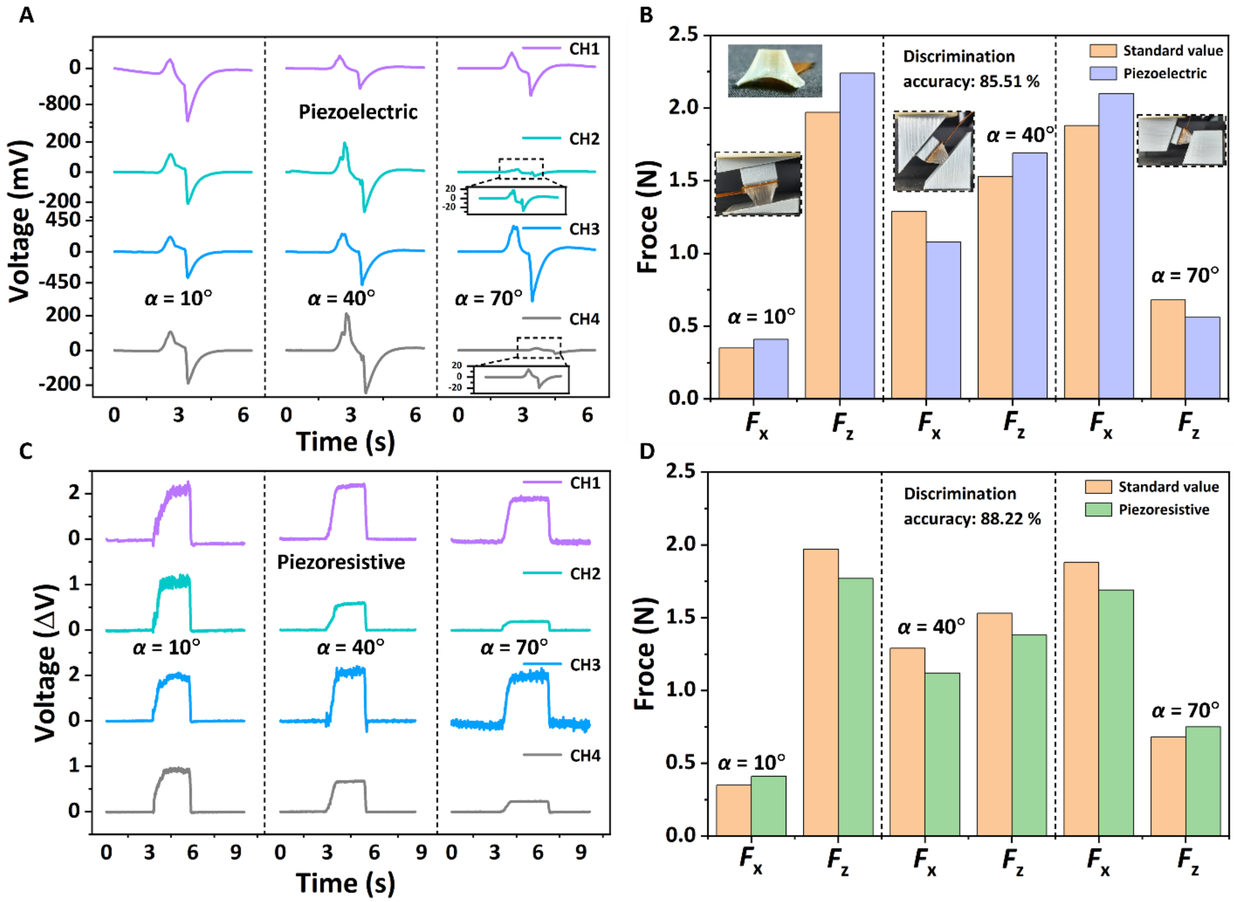

Fig. S13. The decoupling performance of the bimodal haptic sensor in response to variations in mechanical deformations. The real-time output of the (A) piezoelectric and (C) piezoresistive modules. The decoupling accuracies of the (B) piezoelectric and (D) piezoresistive modules.

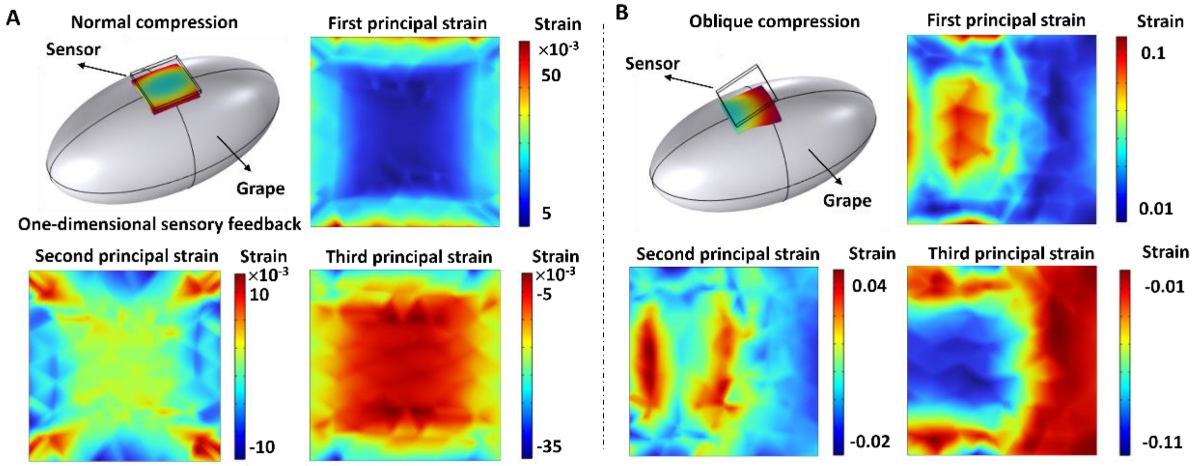

Fig. S14. The deformation of the bimodal sensor. The strain distribution of the piezoelectric sensor in (A) normal and (B) oblique contact with grapes.

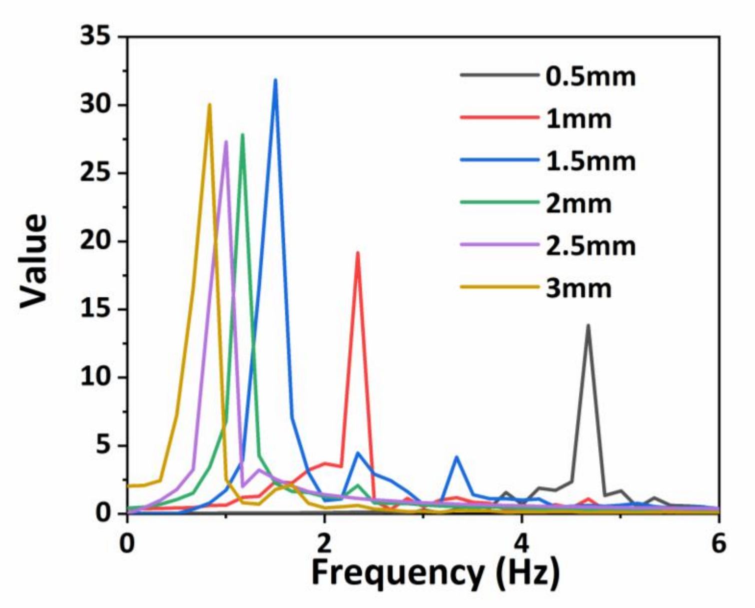

Fig. S15. Fast Fourier transform spectrograms of samples with different spacing.

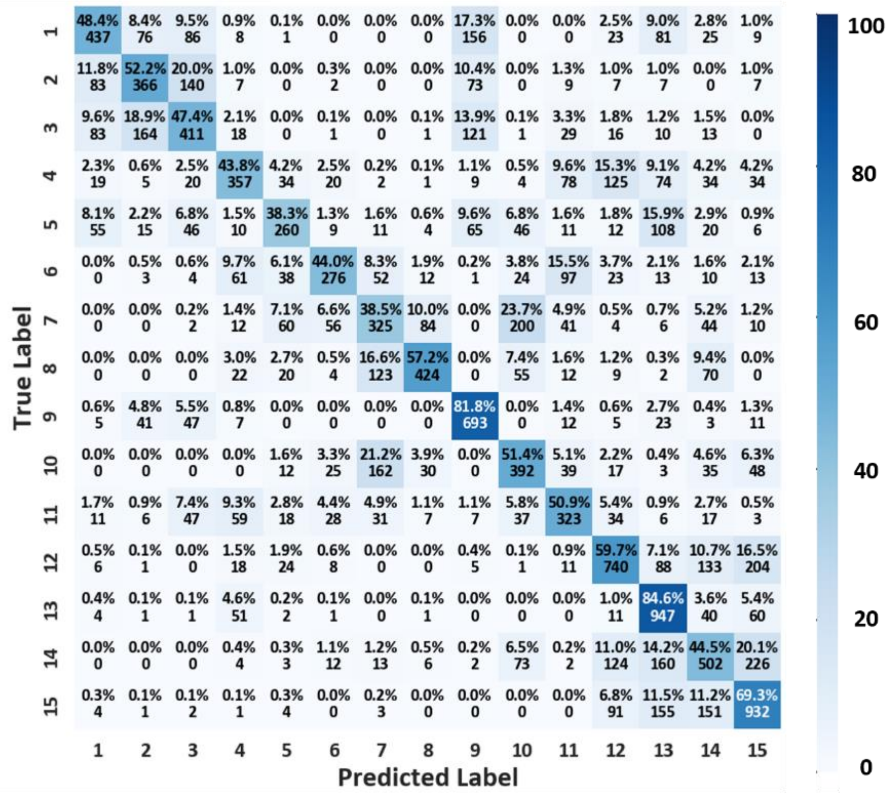

Fig. S16. Confusion matrix for machine learning results on 15 fabrics using one channel feedback.

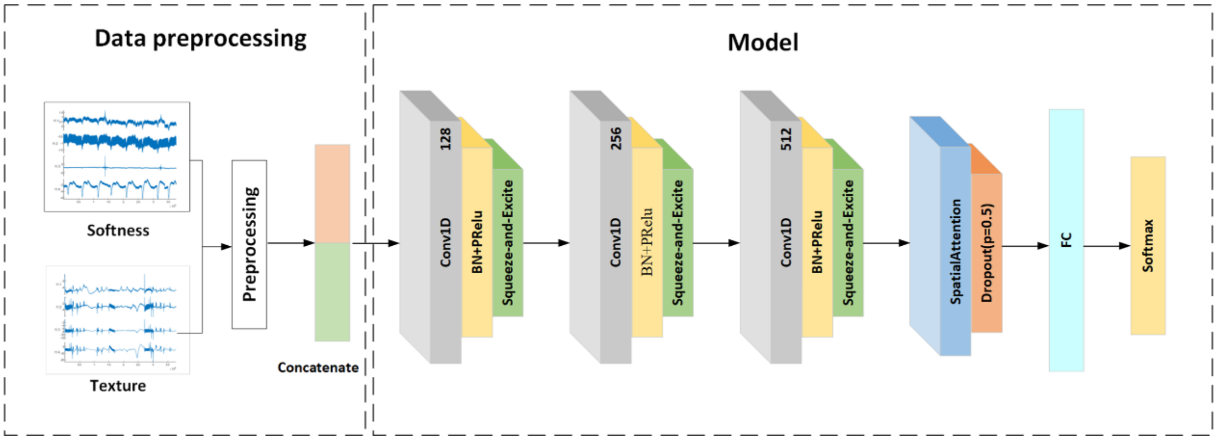

Fig. S17. Schematics of the process and parameters utilized in constructing the artificial neural networks.

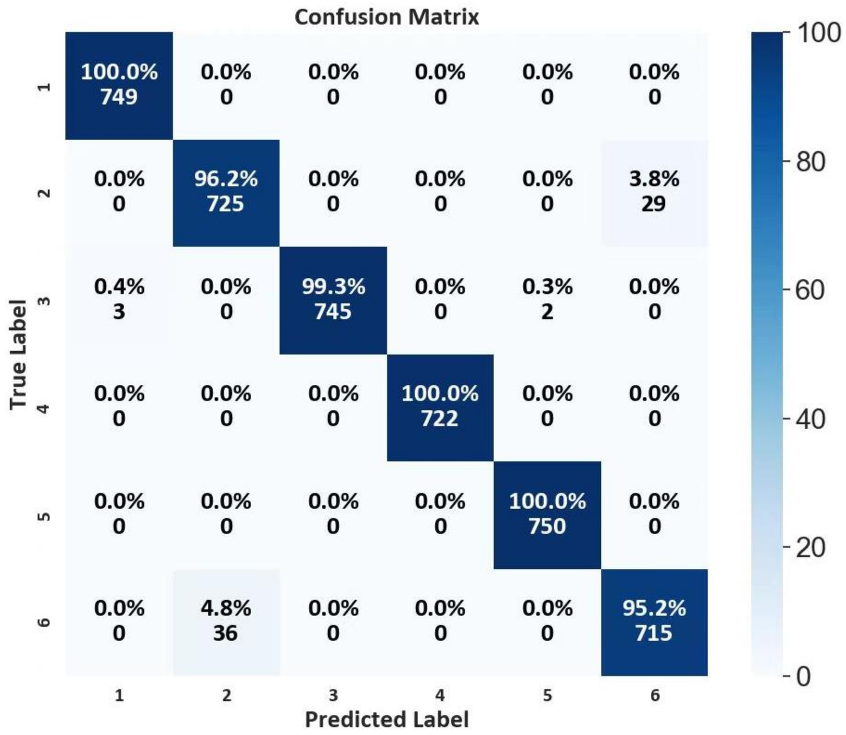

Fig. S18. The confusion matrix for clinical feature identification.

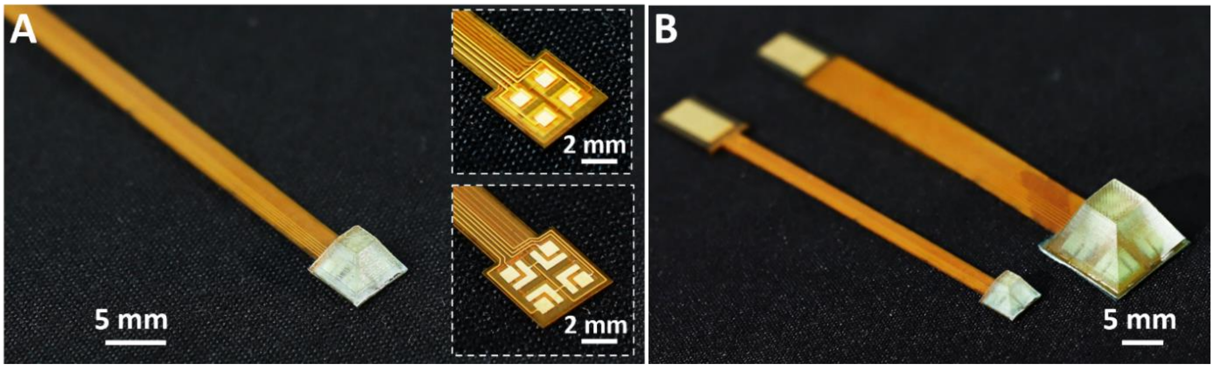

Fig. S19. Optical images of bimodal sensors with different dimensions. (A) The optical image of the bimodal multidimensional sensor in reduced size. (B) Comparison between the reduced-sized sensor and the original-sized sensor.

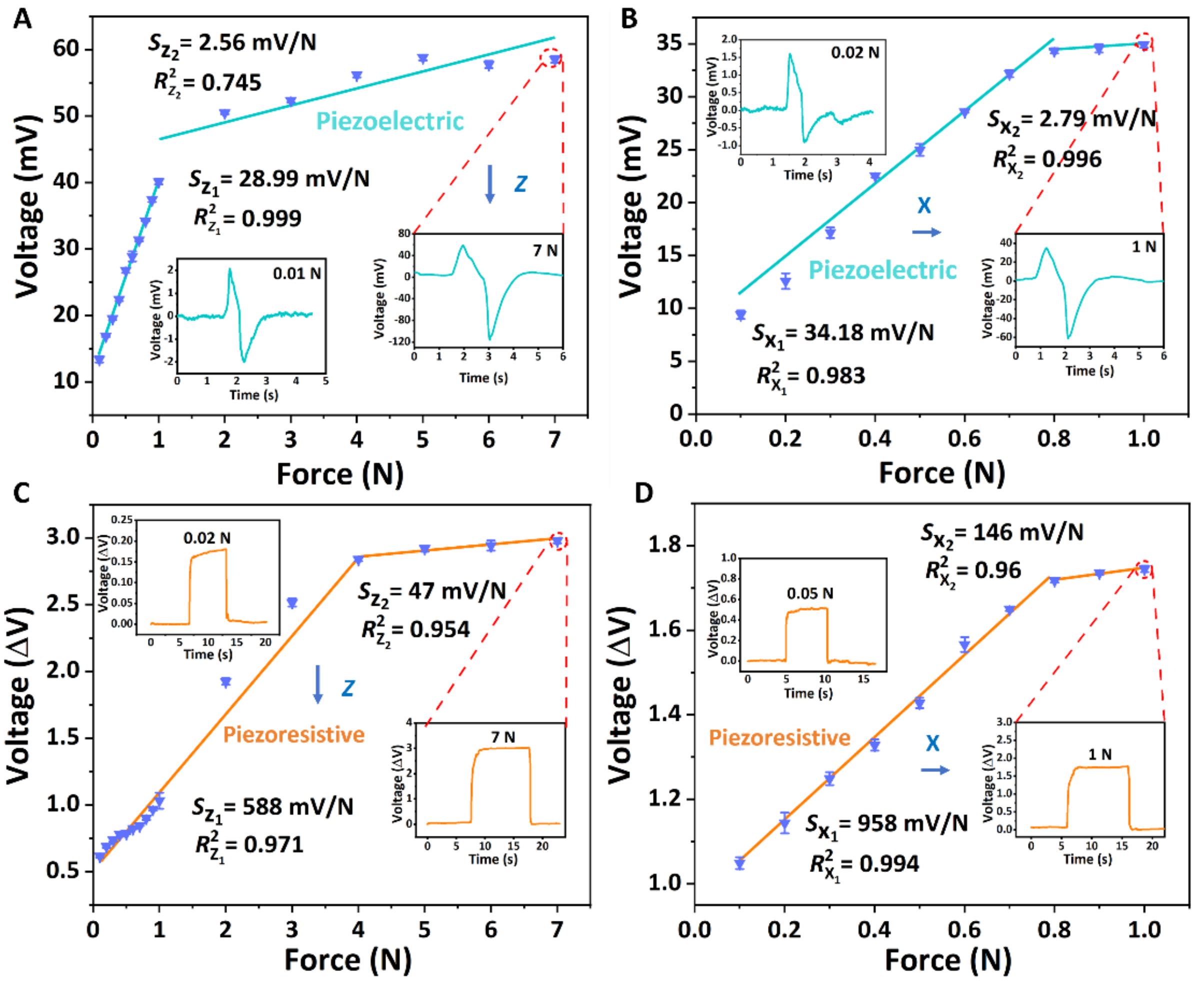

Fig. S20. The sensitivity of the bimodal sensor. The voltage output of sensing modules based on (A, B) piezoelectric and (C, D) piezoresistive mechanisms varies when the applied normal and shear forces range from 0.1 to 7 N. Insets show the measured voltages under the maximum detection limit.

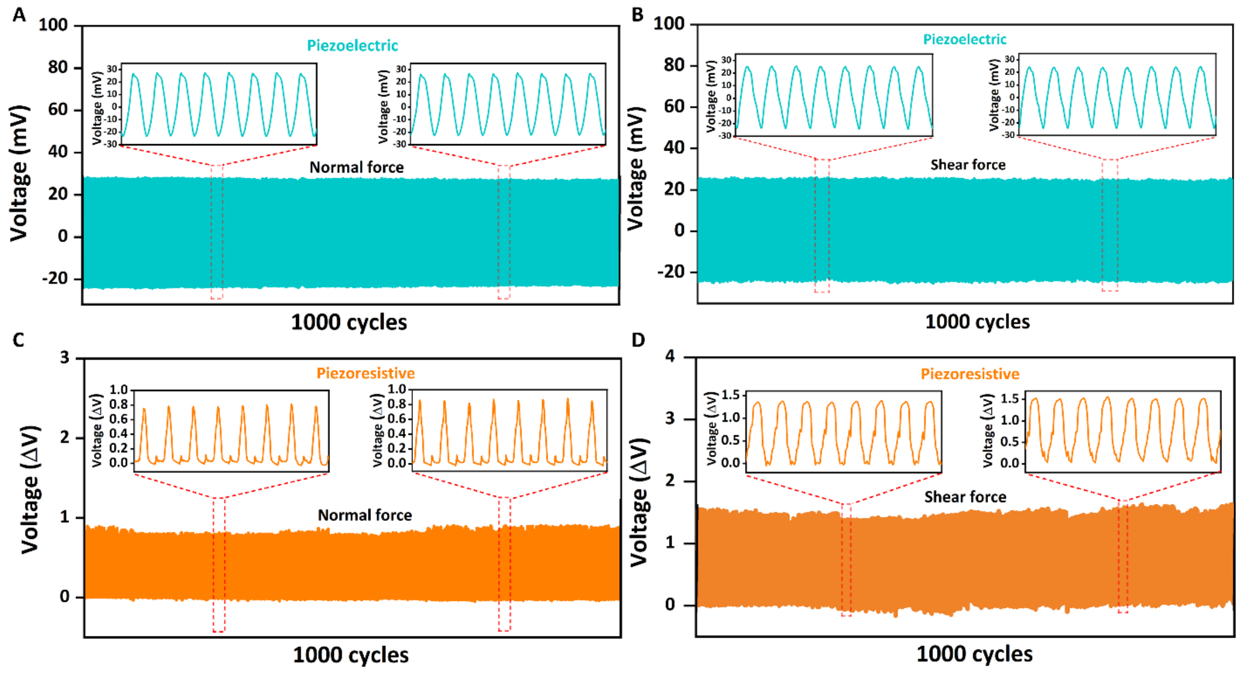

Fig. S21. The sensing stability of the bimodal sensor with small dimensions. Cycling tests of the (A, B) piezoelectric and (C, D) piezoresistive modules tested over 1000 cycles under the normal and shear forces of 0.5 N.

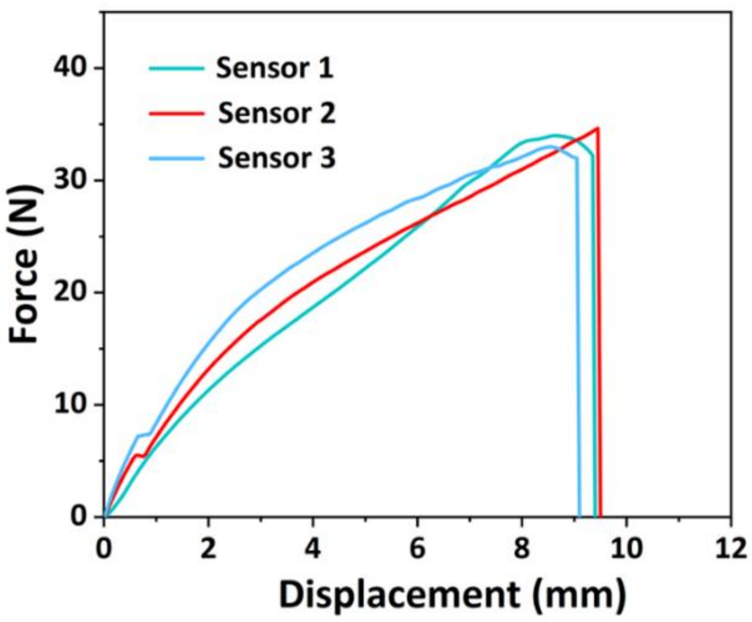

Fig. S22. Force versus displacement curve of PDMS bump on the sensor during peeling test at 0.5 mm/s constant peeling speed.

Table S1. Comparison between the present work and reported tactile sensors.

Sensing Principle	Experimental Maximum sensitivity	Force range	Bandwidth	Force direction detection	Ref.
Piezoelectric	7.32 mV N^{-1}	0.5-4 N	1-120 Hz	Normal	50
Piezoelectric	288 mV N^{-1}	0-25 N	/	Normal	51
Piezoelectric	228.2 mV N^{-1}	0.5-10 N	/	Normal	52
Piezoelectric	0.21 mV N^{-1}	0.7-10 N	1-120 Hz	Normal	53
Piezoelectric	50 mV N^{-1}	0-10 N	10-200 Hz	Normal	54
Piezoelectric	95.8 mV N^{-1}	0-70 N	/	Normal	55
Piezoresistive	0.0256 mV N^{-1}	0-7 N	/	Normal	56
Piezoelectric	46.1 mV N^{-1}	0.5-6 N	/	Normal	57
Piezoelectric	Z: 12.6 ± 0.8 mV N^{-1}<br>XY: 55.2 mV N^{-1}	0.08-0.28 N	1-10 Hz	Normal and shear	58
Piezoresistive	Z: 230 mV N^{-1}<br>X: 650 mV N^{-1}<br>Y: 670 mV N^{-1}	Z: 0-20 N<br>XY: 0-5 N	/	Normal and shear	59
Magnetic	Z: 56.52 mV N^{-1}<br>XY: 50.82 mV N^{-1}	Z: 0-25 N<br>XY: 0-12.5 N	/	Normal and shear	60
Piezoresistive	Z: 160 mV N^{-1}<br>X: 470 mV N^{-1}<br>Y: 450 mV N^{-1}	Z: 0-22 N<br>XY: 0-10 N	/	Normal and shear	61
Piezoelectric	Z: 344.3 mV N^{-1}<br>X: 373.8 mV N^{-1}<br>Y: 414.6 mV N^{-1}	Z: 0.1-2 N<br>X: 0.02-0.44 N	/	Normal and shear	62
Piezoresistive<br>Piezoelectric	Z: 692 mV N^{-1}, $<$1N<br>X: 839 mV N^{-1}, $<$1N<br>Z: 152 mV N^{-1}, ≥1N<br>X: 261 mV N^{-1}, ≥1N	Z: 0.01-35 N<br>X: 0.01-17 N	0-300 Hz	Normal and shear	**This work**

Table S2. Softness classification and elastic coefficients of measured objects.

Measured objects	Softness category	Assigned contact forces (N)	Elastic coefficients of sensors k_1 (N/mm)	Compression deformation of objects x_2 (mm)	Compression deformation of PDMS x_1 (mm)	Elastic coefficients of systems k (N/mm)	Elastic coefficients of objects k_2 obtained by sensing systems (N/mm) (Experimentally measured)
Sponge	A	0.5	8.993	4.47	0.49	0.145	0.147 (0.146)
Foam	A	0.5	8.993	1.64	0.49	0.509	0.540 (0.479)
Ecoflex	B	2	16.64	0.37	0.58	4.829	6.804 (7.551)
EVA	B	2	16.64	0.3	0.58	5.552	8.332 (8.882)
PDMS	C	5	21.115	0.38	0.79	11.496	25.239 (24.416)
PS	C	5	21.115	0.477	0.79	12.97	33.624 (36.453)
Rubber	D	10	23.816	0.09	0.88	15.382	Rigid
Wood	D	10	23.816	0.11	0.88	16.574	Rigid

Table S3. Friction coefficients of measured objects.

Measured objects	Mass+(weight) (g)	Friction forces (g)	Friction coefficients of objects obtained by frictional experiments	Friction coefficients of objects obtained by sensing systems	Error rate
Sponge	1.74+ (10)	10.72	0.91	0.86	-5.8%
Foam	1.12+ (10)	7.18	0.65	0.71	8.5%
Ecoflex	79.42	45.48	0.57	0.62	8.1%
EVA	5.33+ (10)	11.51	0.75	0.72	-4.2%
PDMS	75.11	43.25	0.58	0.62	6.5%
PS	2.53+ (10)	9.89	0.79	0.82	3.7%
Rubber	126.16	72.11	0.57	0.63	9.5%
Wood	31.81	28.49	0.90	0.88	-2.3%

Table S4. The recognition and picking results of white strawberries.

Number of groups	Plant setting-fruit number	Diameter (cm)	Maturity and success (S)/failure (F) situations	Success rate
#1	6	2.19, 3.08, 2.43, 2.27, 3.04, 1.81	ripe (F-rupture), ripe, unripe, unripe, ripe, unripe	83.30%
#2	10	2.29, 2.25, 2.89, 2.81, 2.78, 1.88, 2.3, 2.49, 2.89, 2.88	ripe, ripe (F-recognition), ripe (F-rupture), ripe, ripe, unripe, ripe, ripe, unripe, ripe	80%
#3	9	2.68, 3.27, 2.43, 1.81, 2.09, 3.56, 2.12, 2.75, 3.13	unripe, ripe, ripe, unripe, unripe, ripe, unripe, unripe, ripe	100%
#4	7	1.89, 2.84, 1.7, 2.27, 3.48, 2.69, 2.31	unripe (F-recognition), ripe, ripe, ripe, ripe, unripe, unripe	85.70%
#5	6	2.43, 2.62, 3.11, 2.31, 1.85, 2.01	ripe, ripe, ripe (F-rupture), ripe, unripe, unripe	83.30%
#6	6	2.84, 3.49, 2.85, 2.57, 3.22, 3.56	unripe, ripe (F-rupture), unripe, unripe (F-recognition), ripe, ripe	66.70%
#7	4	3.5, 3.07, 2.99, 2.28	ripe (F-recognition), ripe, ripe, unripe	75%
#8	8	3.19, 2.63, 1.87, 2.34, 2.71, 2.86, 2.56, 1.72	ripe, unripe (F-recognition), unripe, unripe, ripe (F-rupture), ripe, ripe, unripe	75%
#9	3	2.77, 2.53, 1.87	ripe, ripe, unripe	100%
#10	5	3.34, 2.77, 1.96, 2.01, 2.82	ripe, ripe, unripe, unripe, ripe (F-recognition)	80%
#11	5	2.31, 2.32, 2.38, 2.04, 3.18	ripe, ripe, ripe (F-recognition), unripe, unripe	80%
#12	9	1.92, 2.02, 2.09, 2.94, 3.32, 1.91, 1.95, 1.84, 3.33	unripe, unripe, unripe, ripe, unripe, unripe, unripe (F-recognition), unripe (F-recognition), ripe	77.78%
#13	7	1.96, 2.77, 3.55, 3.31, 3.19, 2.87, 2.28	unripe, unripe, ripe, ripe, ripe (F-recognition), ripe, unripe	85.71%
#14	7	3.55, 2.59, 2.78, 2.87, 1.81, 2.56, 2.71	ripe (F-recognition), unripe, unripe, ripe, unripe (F-slip), ripe, ripe	71.43%
#15	4	2.34, 2.38, 2.01, 1.74	ripe, ripe, ripe, ripe	100%
#16	5	2.75, 2.48, 3.12, 2.49, 1.95	ripe, ripe, ripe (F-recognition), unripe, unripe	80%
Overall success rate (total number of strawberries: 101, number of successful attempts: 83)				82.18%

Movie S1. Demonstration of the porcine esophageal features identification.

Movie S2. Demonstration of a robotic manipulator picking unripe and ripe white strawberries without sensing feedback.

Movie S3. Demonstration of the unripe and ripe white strawberries picking procedures utilizing solely softness sensing feedback.

Movie S4. Demonstration of intelligent picking and stable grasping the ripe white strawberry with softness and texture sensing feedback.
